# Digital mindfulness-based intervention for people with COPD – a multicentre pilot and feasibility RCT

**DOI:** 10.1186/s12931-025-03243-4

**Published:** 2025-05-26

**Authors:** Hannah Tschenett, Florian Vafai-Tabrizi, Ralf Harun Zwick, Arschang Valipour, Georg-Christian Funk, Urs M. Nater

**Affiliations:** 1 Department of Clinical and Health Psychology, Faculty of Psychology, University of Vienna, Vienna, Austria; 2 Karl Landsteiner Institute for Lung Research and Pulmonary Oncology, Vienna, Austria; 3 Association for the Promotion of Scientific Research at the Wilhelminen Hospital of the City of Vienna, Vienna, Austria; 4 Research Platform “The Stress of Life (SOLE) - Processes and Mechanisms underlying Everyday Life Stress”, Vienna, Austria; 5 Ludwig Boltzmann Institute for Rehabilitation Research, Therme Wien Med, Vienna, Austria; 6 Department of Respiratory and Critical Care Medicine, Klinik Floridsdorf, Vienna, Austria

**Keywords:** COPD, Digital mindfulness intervention, Feasibility, Anxiety, Depression

## Abstract

**Background:**

Mindfulness-based interventions (MBIs) are effective in improving mental and physical health in various chronic conditions. While the GOLD 2024 report recommends MBIs for chronic obstructive pulmonary disease (COPD), scientific evidence in this specific population is scarce. This prospective randomised controlled pilot study investigated the feasibility of an 8-week digital MBI and its preliminary effects on mental and physical health in COPD.

**Methods:**

Psychologically burdened COPD patients (63 ± 7 years, 61% female, FEV1% 41 ± 19) were randomly allocated to the MBI group (*n* = 14; daily 10-15-minute audio-guided meditation via smartphone) or a waitlist control group (*n* = 16). Primary outcomes included the intervention’s feasibility (dropouts, MBI usage rates, interview and questionnaire responses) and its preliminary effects on symptoms of anxiety and depression (Hospital Anxiety and Depression Scale, HADS). Secondary outcomes included its preliminary effects on the COPD Assessment Test (CAT), Chronic Respiratory Disease Questionnaire (CRQ-SAS), Perceived Stress Scale (PSS-10), and biological stress markers. Exploratory outcomes included momentary subjective stress, anxiety, and dyspnoea after meditating.

**Results:**

The results indicated that the intervention was feasible (81% usage rate; 93% and 71% found the MBI enjoyable and helpful, respectively), with 21% dropout. A statistically significant intervention (time x group) effect was found for anxiety (HADS-A, *p* =.010, *η*_*p*_^*2*^ = 0.11) and emotional functioning (CRQ-SAS, *p* =.004, *η*_*p*_^*2*^ = 0.14), but not for depression (HADS-D, *p* =.060, *η*_*p*_^*2*^ = 0.06) or any other secondary outcome after 8 weeks. Momentary subjective stress (*p* <.001, *η*_*p*_^*2*^ = 0.75), anxiety (*p* =.022, *η*_*p*_^*2*^ = 0.75), and dyspnoea (*p* <.001, *η*_*p*_^*2*^ = 0.70) were significantly reduced after meditating.

**Conclusions:**

The digital MBI was feasible, with preliminary effects indicating improvements in anxiety and emotional functioning after 8 weeks as well as momentary outcomes after meditating. Future large-scale trials should further assess the effectiveness of digital MBIs in this context. However, the findings suggest that digital MBIs might be promising and effective low-threshold add-on treatments in clinical settings.

**Trial registration:**

The article has been preregistered at ClinicalTrials.gov (identifier: NCT04769505, date: 23rd February 2021).

**Supplementary Information:**

The online version contains supplementary material available at 10.1186/s12931-025-03243-4.

## Background

Around a third of patients with chronic obstructive pulmonary disease (COPD) experience clinically significant levels of anxiety [1] and a quarter report clinically significant depressive symptoms [2]. While both negatively impact other health-related outcomes, including physical health impairment and health-related quality of life [3], they often remain undiagnosed and untreated. Furthermore, biological correlates and psychological symptoms of stress are closely linked to symptoms of anxiety and depression in other somatic conditions [4, 5] but are largely overlooked in COPD. Patients with COPD are repeatedly or even continuously exposed to stressors such as breathing problems [6]. Moreover, the dysregulation of bodily stress systems (hypothalamic-pituitary-adrenal (HPA) axis, autonomic nervous system (ANS), immune system), reflected by alterations in their basal activity or reactivity to stressors [7], increases the body’s effort to maintain homeostasis, resulting in chronic stress [8]. In COPD, elevated systemic inflammation and general sympathetic overactivity of the ANS [9, 10] may indicate such dysregulations and associated chronic stress.

Although the 2024 GOLD international practice guidelines recommend stress management strategies and mind-body interventions for the management of dyspnoea and mental health symptoms [11], evidence on the effectiveness of these interventions in COPD is scarce and mixed [12, 13]. This underlines the urgency of research on feasible and effective psychological interventions for COPD patients which are easy to administer and conduct and can be implemented in clinical settings and patients’ everyday life. Mindfulness-based interventions (MBIs) are mind-body interventions that train present-moment awareness through different activities (e.g., meditation) and contain psychoeducational elements [14]. They are effective in reducing anxiety and depressive symptoms, self-reported stress [15], and biological stress markers [16], and in improving physical health outcomes in somatic conditions [17]. Digitally delivered MBIs are likewise effective [18] and might be particularly promising for COPD patients, as they can be self-administered, are accessible to non-mobile patients, easy-to-learn, cost-effective, and can be implemented in both clinical settings and everyday life [19]. They also allow for tailored adaptations regarding patients’ needs (e.g., brief activities) and are acceptable for patients [20], potentially enhancing treatment adherence. Although many MBI apps for COPD patients are available [21], evidence on the effects of these self-administered digital MBIs for COPD patients is lacking.

## Methods

### Study design

In our research project, we follow the framework for developing and evaluating complex health interventions by the UK Medical Research Council [22]. This framework aims to support the implementation of interventions that are not only effective but also “acceptable, implementable, cost-effective, scalable, and transferable across contexts” (p. 2 [22]). It outlines four key steps: (1) identifying or developing a potentially suitable and beneficial intervention, (2) testing its feasibility, (3) evaluating its effectiveness, and (4) facilitating its implementation. Based on findings from a previous study that identified the need for and potential of a digital MBI for individuals with COPD [20], we developed the intervention examined in this pilot study (step 1). The current pilot randomised controlled trial corresponds to step 2 of the framework. Its objective was to assess the feasibility of an 8-week self-administered digital MBI to identify potential challenges and adaptation criteria for a future large-scale randomized controlled trial (step 3), and to gather preliminary data on the intervention’s effects to inform power calculations and determine which observed trends may be promising for further investigation in a future definitive trial (based on recommendations for pilot studies [22–24]).

The primary outcome was feasibility among individuals with COPD and elevated levels of anxiety or depression by examining (a) dropout rates, (b) usage behaviour, (c) patients’ experiences as reported in telephone interviews after 8 weeks, and (d) long-term treatment adherence after 6 months. Further, we aimed to assess the intervention’s preliminary effects and hypothesised that the MBI may reduce levels of anxiety and depression after 8 weeks. Regarding secondary outcomes, we hypothesised reduced self-reported stress, lower physical health impairment, and improved COPD-related quality of life in the intervention group after 8 weeks. We further investigated basal activity and stress reactivity of two bodily stress systems (ANS and HPA axis) to explore possible changes in these systems’ functioning due to the MBI. We therefore hypothesized lower hair cortisol levels (correlate of basal HPA axis activity reflecting cumulative cortisol secretion over several months [7]), and improved stress reactivity on a subjective and biological level (assessing heart rate, heart rate variability, and skin conductance level as correlates of ANS reactivity [7]) in the intervention group after 8 weeks. Finally, we investigated momentary subjective stress, anxiety, and dyspnoea immediately before and after the individual MBI activities from an exploratory perspective.

**Fig. 1:**
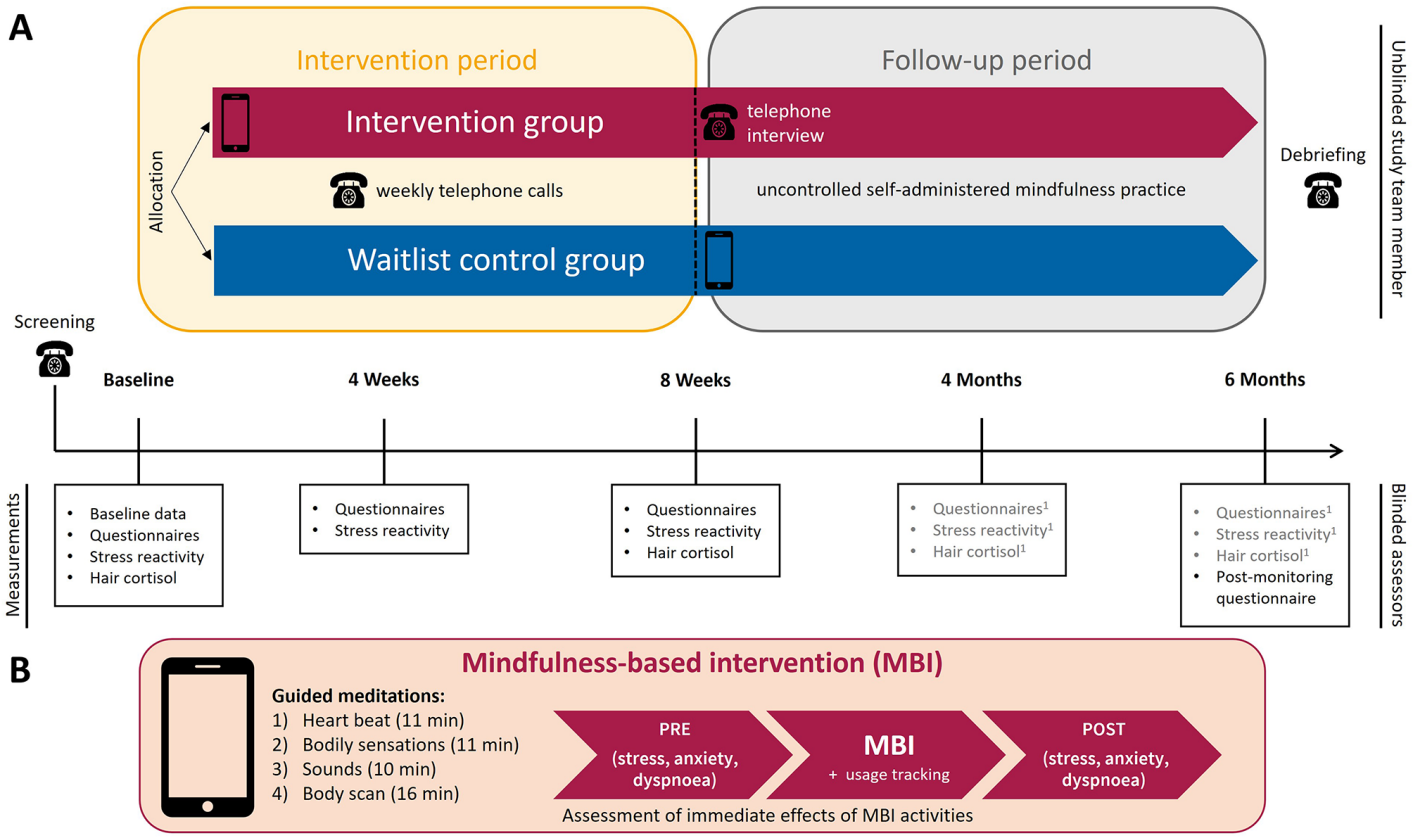
Study overview. *Note.***(A)** Study timeline. Measurements were conducted in patients’ homes (see Suppl. 1 for COVID-19 protocol to ensure safe in-person contact) to include non-mobile patients, unless patients explicitly preferred assessments in the laboratory at the University of Vienna. ^1^These data are not relevant to the scope of this publication. **(B)** Mindfulness-based intervention (MBI). Icons provided by www.flaticon.com

An overview of the study is provided in Fig. 1. The study was in accordance with the Declaration of Helsinki, approved by the city of Vienna ethics review board (serial number: EK 20–177 VK), and preregistered at ClinicalTrials.gov (identifier: NCT04769505).

### Participants

**Fig. 2:**
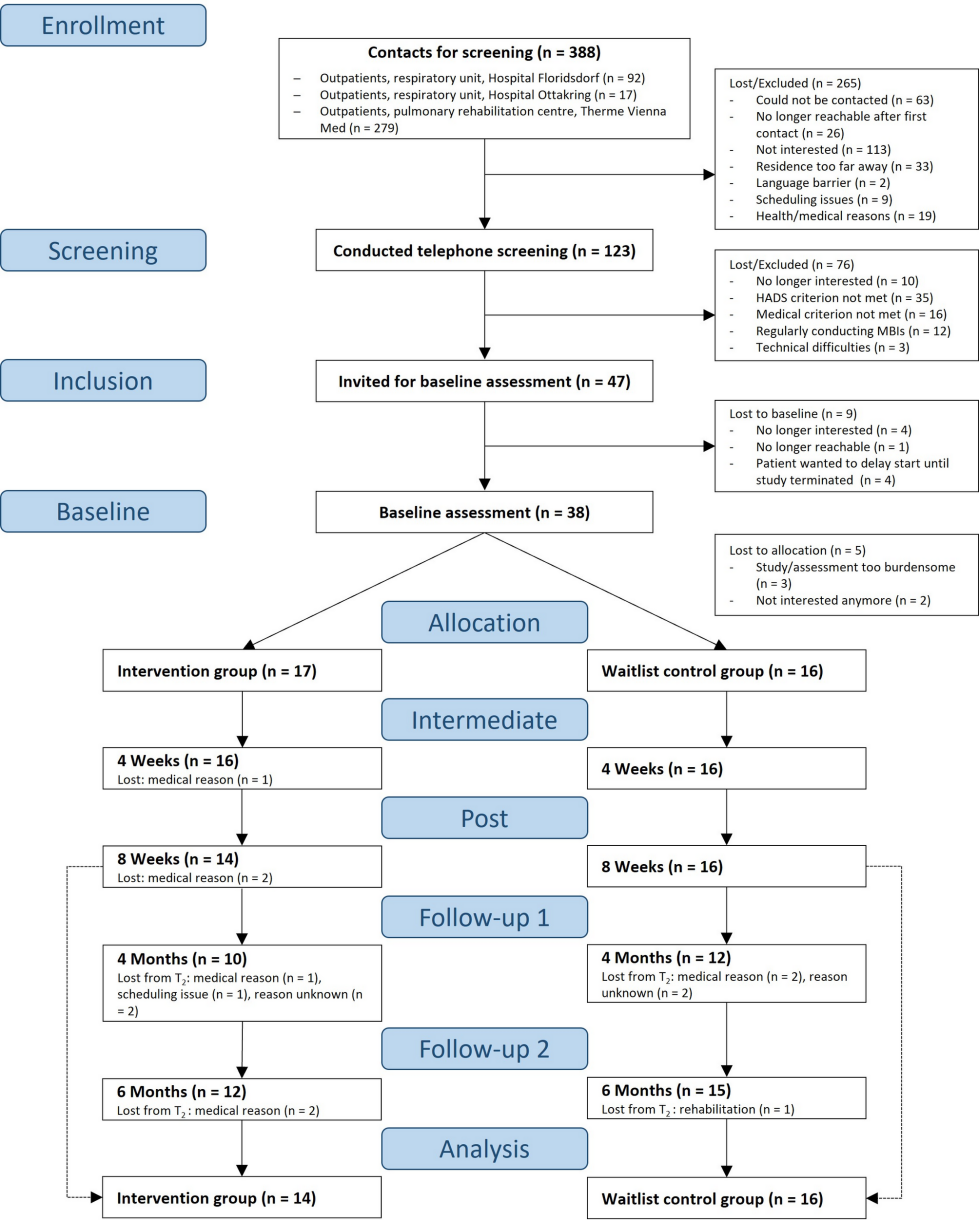
Study flow and recruitment diagram. *Note.* Following the study preparation phase (September 2020 – March 2021), patients were recruited from March 2021 – December 2022, until 30 participants concluded the intervention period. Assessments lasted from March 2021 – March 2023

We aimed to recruit 30 COPD patients (*n* = 15 per treatment arm) completing the intervention period, based on the sample size recommendations for pilot studies aiming at a 90%-powered main trial [24], assuming a medium effect size (0.3 ≤ *d* ≤ 0.7) for symptoms of anxiety, depression, and stress for an 8-week intervention effect [15]. According to the CONSORT extension to pilot studies, the sample size should be determined based on the feasibility objective [23]. Our sample size is adequate to determine feasibility, consistent with studies using similar methods (e.g. sample range of 5–32 for qualitative meta-synthesis by [13]). Outpatients with stable COPD from two clinics and a rehabilitation centre were contacted for screening (see Fig. 2 for details on recruitment and study flow). Eligible patients (see Table 1 for eligibility criteria) received detailed information about study participation and provided written informed consent at baseline. Baseline participant characteristics are summarised in Table 2 (for full baseline characteristics, see Table S1).

**Table 1:** Inclusion and exclusion criteria

Inclusion criteria		Exclusion criteria	
a	spirometry-confirmed COPD diagnosis (forced expiratory volume (FEV) 1 in percent < 80%)	a	active asthma diagnosis
b	≥ 40 years	b	AECOPD within the last month
c	sufficient German language skills	c	severe auditory impairments
d	physically and mentally capable of participating	d	severe comorbid psychiatric disorder (e.g., severe cognitive impairments, psychotic or associated disorders)
e	psychologically burdened (defined by a cut-off ≥ 8 for subclinical symptoms of anxiety or depression on the corresponding subscale of the Hospital Anxiety and Depression Scale [25], HADS-A or HADS-D)	e	severe comorbid somatic disorder (e.g., heart failure (LVF < 35%, unstable coronary heart disease, uncontrolled diabetes, active cancer, stroke, ventilated patients with lung failure)
f	capable of using a (study) smartphone for the intervention	f	active infection or health issue interfering with study participation (e.g., infection with SARS-Cov-19)
g	smoke-free for the duration of the assessments (up to 4 h)	g	regular (at least once a week) psychosocial support (e.g., psychotherapy)
h	residency in or the surrounding areas of Vienna (≤ 1 h car drive from the city centre of Vienna)	h	regular (at least once a week) mind-body practice (e.g., yoga, autogenic training)
		i	participation in another clinical trial

Note. Participants were excluded during their study participation if any exclusion criterion changed or if participants had been hospitalised. All eligibility criteria are based on patients’ self-report, with the exception of inclusion criterion (a), which is based on medical records, and inclusion criterion (c), which is based on the screener’s assessment. FEV = forced expiratory volume, HADS = Hospital Anxiety and Depression Scale, AECOPD = acute exacerbation in COPD, LVF = left ventricular function

**Table 2:** Selected baseline characteristics

	Intervention (*n* = 17)		Waitlist (*n* = 16)		Total (*N* = 38)	
	*n*	%	*n*	%	*n*	%
Age (*M* ± *SD*; Range)	62.59 ± 7.75; 47–77		62.06 ± 5.93; 53–72		62.68 ± 7.07; 47–77	
Gender						
Male	8	47.1	5	31.3	15	39.5
Female	9	52.9	11	68.8	23	60.5
Native language						
German	14	82.4	15	93.8	31	81.6
Other	3	17.6	1	6.3	7	18.4
Family status						
Single/divorced/widowed	9	52.9	7	43.8	19	50
Married/partnered	8	47.1	9	56.3	19	50
Education						
Lower secondary school	5	29.4	3	18.8	8	21.1
Apprenticeship	4	23.5	7	43.8	12	34.2
Grammar school	4	23.5	4	25	9	23.7
College	2	11.8	1	6.3	4	10.5
Bachelor/Master	2	11.8	1	6.3	4	10.5
Employment						
(Self-)Employed	1	5.9	5	31.3	6	15.8
Retired	13	76.5	10	62.5	26	68.4
Unemployed	3	17.6	1	6.3	6	15.8
Receiving care	7	41.2	3	18.8	12	31.6
BMI (*M* ± *SD*; Range)	25.45 ± 5.97; 17–38		24.06 ± 2.62; 19–28		25.14 ± 4.75; 17–38	
Duration COPD in years (*M* ± *SD*; Range)	9.12 ± 5.28; 1–20		10.47 ± 5.50; 5–23		10.46 ± 6.13; 1–23	
FEV1 Liter (M ± SD; Range) (*n* = 36)	1.06 ± 0.67; 0–3		1.14 ± 0.59; 1–2		1.18 ± 0.67; 0–3	
FEV1% (M ± SD; Range) (*n* = 36)	35.46 ± 16.29; 18–77		41.8 ± 19.79; 20–82		41.21 ± 19.16; 18–82	
FEV1/FVC Percent (*M* ± *SD*; Range) (*n* = 35)	45.44 ± 15.26; 30–85		43.26 ± 13.16; 28–66		46.99 ± 14.94; 28–85	
COPD Stage (*n* = 36)						
I	1	5.9	0	0	1	2.8
II	3	17.6	1	6.7	4	11.1
III	2	11.8	9	60.0	14	38.9
IV	11	64.7	5	33.3	17	47.2
Medical conditions						
Previous	1	5.9	3	18.8	4	10.5
Current (acute or chronic)	8	47.1	5	31.3	17	44.7
Mental disorder						
Previous	2	11.8	2	12.5	5	13.2
Current (acute or chronic)	2	11.8	3	18.8	6	15.8
Number of prescribed medications (*M* ± *SD*; Range)	6.35 ± 2.57; 4–12		5.25 ± 3.55; 0–12		5.74 ± 3.12; 0–12	
Treatments for COPD						
LAMA	15	88.2	15	93.8	34	89.5
LABA	16	94.1	14	87.5	33	86.8
ICS	15	88.2	12	75	29	76.3
Roflumilast	2	11.8	3	18.8	5	13.2
Bronchodilator	13	76.5	10	62.5	25	65.8
Oxygen therapy	10	58.8	7	43.8	19	50
Pulmonary rehabilitation (*n* = 37)	13	76.5	14	93.3	32	86.5
Smoking (*n* = 37)						
No	1	5.9	0	0	1	2.7
Previous	13	76.5	10	66.7	24	64.9
Current	2	11.8	3	20.0	9	24.3
Current (very reduced or changed substance)	1	5.9	2	13.3	3	8.1
Pack years (*M* ± *SD*; Range) (*n* = 36)	45.88 ± 31.04; 11–100		44.57 ± 20.61; 15–84		42.83 ± 25.92; 6–100	
Previous contact psych. professionals						
Yes	10	58.8	12	75	25	65.8
No	7	41.2	4	25	13	34.2
Previous experience mind-body interventions						
Yes	12	70.6	10	62.5	25	65.8
No	5	29.4	6	37.5	13	34.2
Patient Health Questionnaire (PHQ)^1^						
Somatic symptoms (PHQ-15) (*M* ± *SD*; Range) (*n* = 35)	10.82 ± 5.87; 2–24		9.00 ± 4.00; 3–16		10.17 ± 5.00; 2–24	
Depression (PHQ-9) (*M* ± *SD*; Range) (*n* = 36)	7.35 ± 4.23; 0–13		8.63 ± 3.88; 3–16		7.83 ± 3.95; 0–16	
Stress (*M* ± *SD*; Range) (*n* = 30)	5.85 ± 3.18; 1–12		5.69 ± 3.43; 1–13		5.73 ± 3.06; 1–13	
Somatoform disorder (*n* = 37)	7	41.2	5	31.3	14	37.8
Major depressive disorder	0	0	2	12.5	2	5.3
Other depressive disorder	4	23.5	5	31.3	10	26.3
Panic disorder (*n* = 37)	1	5.9	3	20	4	10.8
Other anxiety disorder	1	5.9	0	0	1	2.6
Alcohol disorder (*n* = 37)	3	18.8	5	31.3	8	21.6

Note. Analysed data from all *N* = 38 participants who completed the baseline assessment. *n* = 5 dropped out prior to treatment allocation. Percent are valid percent for the sample without missing values. In the case of missing values, the *n* for the valid percent is reported in brackets. BMI = body mass index, FEV = forced expiratory volume, FVC = forced vital capacity, LAMA = long-acting muscarinic antagonist, LABA = long-acting beta antagonist, ICS = inhaled corticosteroids. 1: The PHQ is a validated screening tool for mental health and was administered at baseline only to characterize the sample regarding their mental health [44]

### Randomisation and blinding

Following a baseline session, patients were randomised to the intervention (MBI plus treatment as usual (TAU)) or waitlist control group (TAU) using block randomisation (allocation ratio 1:1; random block lengths of 2, 4, and 6), stratified by patients from the respiratory unit Floridsdorf on a waitlist for a bronchoscopic lung volume reduction (as these had been preselected regarding their eligibility for the procedure) and all other patients. The random treatment allocation was generated for each sub-sample by one study team member prior to data collection. After baseline, the same study team member informed the patient by telephone about the treatment allocation. The assessors conducting the recruitment and measurements were blinded regarding treatment allocation. The assessor blinding was broken during 26 measurements (23%), as patients articulated their treatment allocation.

### Treatment

The four auditory-guided meditations (for details see Fig. 1) are based on mindfulness-based cognitive therapy [26] and its adaptations for COPD patients (e.g., heartbeat as an anchor) by Farver-Vestergaard and colleagues [27] and were further adapted for this study (e.g., shortened, simpler language). The audios were delivered via the movisensXS software (Movisens GmbH) installed on patients’ (study) smartphones. Patients received an individual face-to-face introductory session (30–50 min) and were instructed to practice at least once daily during the intervention period. Additionally, an intervention manual (Supplementary Material 2 at https://osf.io/sx5h9/) and a weekly telephone call supported their self-administered mindfulness practice. After 8 weeks, the intervention group received the MBI meditations as audio files with the instruction to continue practicing in the follow-up period.

Waitlist patients received the MBI after the intervention period, with the same instructions, manual, and introductory session as the intervention group. However, the meditations were provided directly as audio files. To control for the psychological support provided by the weekly telephone calls, the study team also called the waitlist group weekly during the intervention period to discuss health status and experiences of the past week.

### Measures

#### Primary outcomes

To assess feasibility, the dropout rate was defined as the number of patients who started the study but dropped out before concluding the intervention period. Usage behaviour was defined as the number of meditations (≥ 10 min) conducted by the intervention group within the intervention period, using log numbers from the study software. The telephone interviews with intervention group participants after 8 weeks assessed the acceptability and usability of the intervention and participants’ satisfaction using and open-ended entry question, closed questions, and open-ended follow-up questions (for details see Suppl. 3 and Supplementary Material 1 at https://osf.io/sx5h9/). Interviews were conducted by one study team member and were audiotaped. The structured post-monitoring questionnaire assessed long-term adherence to the self-administered mindfulness practice in the follow-up period using three dichotomous items (“Did you conduct mindfulness activities during the last 4 months?”, “Did you use the study mindfulness activities during the last 4 months?”, “During the past 4 months, have you performed other mind-body techniques such as relaxation exercises, meditation, autogenic training, imaginative processes, yoga, qi gong, or tai chi?”, for details see Suppl. 4).

Symptoms of anxiety and depression were assessed at three measurement time points (baseline, 4 weeks, 8 weeks) using the corresponding subscales and sum score of the Hospital Anxiety and Depression Scale (HADS) [25]. Sum scores for each scale range from 0 to 21, with higher scores representing higher symptom burden (Anxiety, HADS-A: *α* = 0.71, Depression, HADS-D: *α* = 0.80).

#### Secondary outcomes

The Perceived Stress Scale (PSS-10) [28] was used to assess self-reported ongoing stress at three measurement time points (baseline, 4 weeks, 8 weeks). Sum scores range from 0 to 40, with higher scores indicating higher stress (*α* = 0.82). Physical health impairment was assessed at three measurement time points (baseline, 4 weeks, 8 weeks) using the COPD Assessment Test (CAT) [29]; sum scores range from 0 to 40, with higher scores indicating greater impairment (*α* = 0.80). COPD-related quality of life was assessed at three measurement time points (baseline, 4 weeks, 8 weeks) using the Chronic Respiratory Questionnaire Self-Administered Standardized (CRQ-SAS)^1^ [30], with items rated from 1 to 7, covering four domains: dyspnoea (*α* = 0.85), fatigue (*α* = 0.81), emotional function (*α* = 0.80), and mastery (*α* = 0.73).

Hair samples were collected at two measurement time points (baseline, 8 weeks) to analyse cumulative hair cortisol concentration (pg/mg) from the last two months (2-cm segments of scalp-near hair). Analyses were conducted by the biochemical laboratory at the Faculty of Psychology, University of Vienna, following the analysis protocol by Stalder and colleagues [31].

Stress reactivity was measured at three measurement time points (baseline, 4 weeks, 8 weeks) during a stress induction protocol using a cognitive stressor (modified Stroop task, based on [32, 33], adapted for this study; for details see Suppl. 2 including Figure S1). Momentary subjective stress was assessed at five time points (5 min before Stroop, before Stroop, after Stroop, 5 min after Stroop, 10 min after Stroop) during the stress induction protocol, using a unipolar visual analogue scale (“At the moment I feel stressed”) ranging from 0 (*not at all*) to 100 (*very much*). Stress-related ANS reactivity markers (heart rate (HR), root mean square of successive difference (RMSSD) indicating heart rate variability, and skin conductance level, SCL) were continuously measured during the stress induction protocol, using Movisens sensors (Movisens GmbH). Stress reactivity was calculated as the difference score between the time point 5 min before and right after the Stroop task for momentary subjective stress, and between the 5 min before and the Stroop task interval for ANS markers (HR, RMSSD, SCL), ensuring that all measures reflect the change from the initial resting state to peak stress exposure. Details regarding biomarker assessments are described in Suppl. 5.

#### Exploratory outcomes

To assess the immediate effects of meditation, patients reported their momentary subjective stress, anxiety, and dyspnoea via the study software before and after each meditation in the intervention period. Momentary subjective stress was assessed on a visual analogue scale (item description in the previous paragraph). Anxiety was assessed using an adapted HADS item (“I feel anxious”) with four response options ranging from 0 (*not at all*) to 3 (*very*). Dyspnoea was measured using a modified Borg Scale [34] with twelve response options ranging from 0 (*no breathlessness at all*) to 10 (*maximal breathlessness*).

### Data analysis

Descriptive statistics were calculated using Excel (Microsoft 365) and IBM SPSS (Version 28.0.1.1). Telephone interviews were fully transcribed semantically by a project assistant. The answers to the closed interview questions were noted by the interviewer and verified using the transcripts. Answers to the open-ended questions were qualitatively analysed by the interviewer following the steps of reflexive thematic analysis (software: MAXQDA Plus, Version 22.2.0) [35]. Primary and secondary analyses comparing groups over time were analysed using linear mixed models. This deviates from our preregistered analysis using repeated measures ANOVAs, as linear mixed models are the recommended state-of-the-art analysis approach for our data structure [36]. We specified two-level models (measurements nested within participants), with the factors time (baseline, 4 weeks, 8 weeks), group (intervention, waitlist), and crossed factors time x group (intervention effect), using restricted maximum likelihood estimation and the Kenward-Roger approximation. Outliers were not excluded from the analysis, with the exception of eight RMSSD outliers that were due to technical issues. Number of medications was added to the models assessing biological outcomes as a level-2 grand-mean centred covariate [37]. Exploratory analyses were conducted using a two-level linear mixed model with the factor time (pre, post meditation), restricted maximum likelihood estimation and the Kenward-Roger approximation. Effect sizes were described as partial eta-squared (*η*_*p*_^*2*^) calculated from F-tests. All linear mixed models were calculated in R (Version 4.3.0). Details on data analysis and data preprocessing of the biodata are described in Suppl. 6. Sensitivity analyses (removing outliers, including/removing covariates, calculating a robust model with the R package robustlmm [38]) were conducted to gain additional insights into the data (see Suppl. 7). However, given the small sample size, all results regarding intervention effects should be considered preliminary.

## Results

### Primary outcomes

There were 21.05% dropouts during the intervention period. Dropouts reported significantly fewer pack-years (*t*(34) = 2.16, *p* <.05) and lower intake of inhaled corticosteroids (ICS) (*χ*^2^(1, 38) = 8.45, *p* <.01) than completers. There were no other significant differences in baseline characteristics between dropouts and completers. At baseline, participants in the intervention and control group did not differ significantly in baseline characteristics or any outcome measures, except for dyspnoea (see Table S4 for baseline comparisons).

On average, participants practiced mindfulness on 81.38% of the 56 intervention days. The usage rates of the 638 conducted meditations were relatively balanced (25.39% heartbeat, 26.18% bodily sensations, 24.76% sounds, 23.67% body scan), with strong intraindividual variations in usage patterns. Ratings of how much participants liked the meditation were consistently good (3.00 ± 1.21 heartbeat, 2.83 ± 0.83 bodily sensations, 2.91 ± 1.14 sounds, 2.59 ± 1.07 body scan, scale from 0 to 4), though individual preferences varied.

**Fig. 3:**
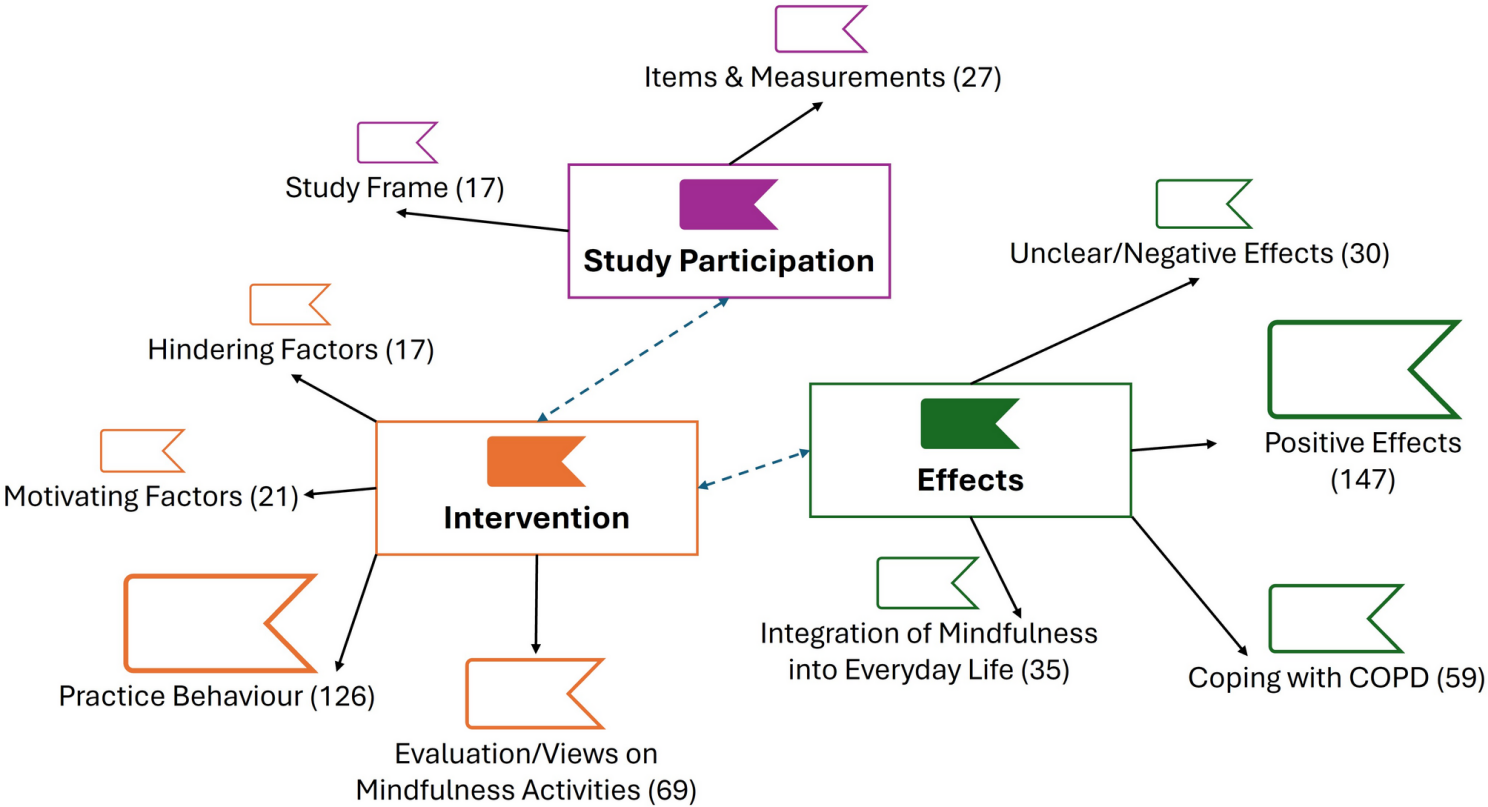
Thematic map of telephone interview. *Note.* Analysed data form *n* = 14 intervention group participants who concluded the intervention period. Thematic map depicts themes (bold) and subthemes identified in the telephone interview assessing feasibility. Numbers in brackets represent the code frequency of each subtheme

Overall, most intervention group participants found the meditations helpful and enjoyable (see Table S2 for descriptive results on the answers to the closed questions of the telephone interview), while also reporting individual preferences (e.g., desire for more meditation variety) during the telephone interviews (see Fig. 3 for a thematic map on the answers to the open-ended questions). For example, one participant mentioned “Because then it’s always the same four topics, it becomes so boring. […] More variety would be better” (ID 30). Participants reported that the intervention was easy to use (“I found it [the intervention] all very easy,” ID 21) and stated no need for additional technical support. The majority reported a successful integration into daily routines (“It’s definitely a routine. […] I always have to take it [medication] in the morning and if I do the exercise in between, I can stretch it [the time between medications] out a bit. […] And it’s better for your digestion,” ID 07). Participants also reported some hindering (e.g., health- and time-related problems) and motivating factors (e.g., commitment, experiencing positive effects) for their practice. For example, one participant mentioned: “Yeah, some days when there was a lot to do at work and also privately, things that needed to be taken care of. Then I was already exhausted, and on top of that, there was [the mindfulness] practice, which felt like an additional task. And on that day, let’s say, it was just one task too many” (ID 38). Another participant described their motivation as follows: “Yes, and what also motivated me was that I felt a bit calmer afterward” (ID 8). Most participants noted changes through practicing mindfulness, mostly referring to positive effects (e.g., relaxation), such as: “I was a bit calmer and more relaxed when I got up and got on with my day” (ID 4). Some also noted changes in coping with COPD (e.g., increased symptom awareness, greater acceptance of COPD symptoms, taking more breaks when breathless). For example, one participant mentioned “As I said, this minor [respiratory] crisis gave me the feeling that I had taken it [the crisis] easier (…) [than] before I did these exercises” (ID 8), while another participant said “I’m not so hectic anymore. Everything in peace. Not quickly, because I used to get up quickly and (…) I can’t do it anymore because of the air” (ID 41). The last quotation also represents a transfer of mindfulness to daily life (e.g., increased calmness and awareness), which some participants mentioned. However, others reported no positive or only short-term effects, particularly regarding respiratory symptoms. One example would be “I had the feeling that I just couldn’t concentrate, that I couldn’t switch off. […] So I had the feeling that it [intervention] had no effect” (ID 37).

While almost all intended to continue practicing and about three quarters wanted to extend their mindfulness practice, only four of twelve participants reported practicing during the follow-up period in the post-monitoring questionnaire (see Table S3 for complete results). Among these, all used the study exercises and one person additionally incorporated other mind-body activities. In contrast, all waitlist group participants practiced mindfulness during the follow-up period, mostly using study activities, with two participants also incorporating other mind-body activities.

**Fig. 4:**
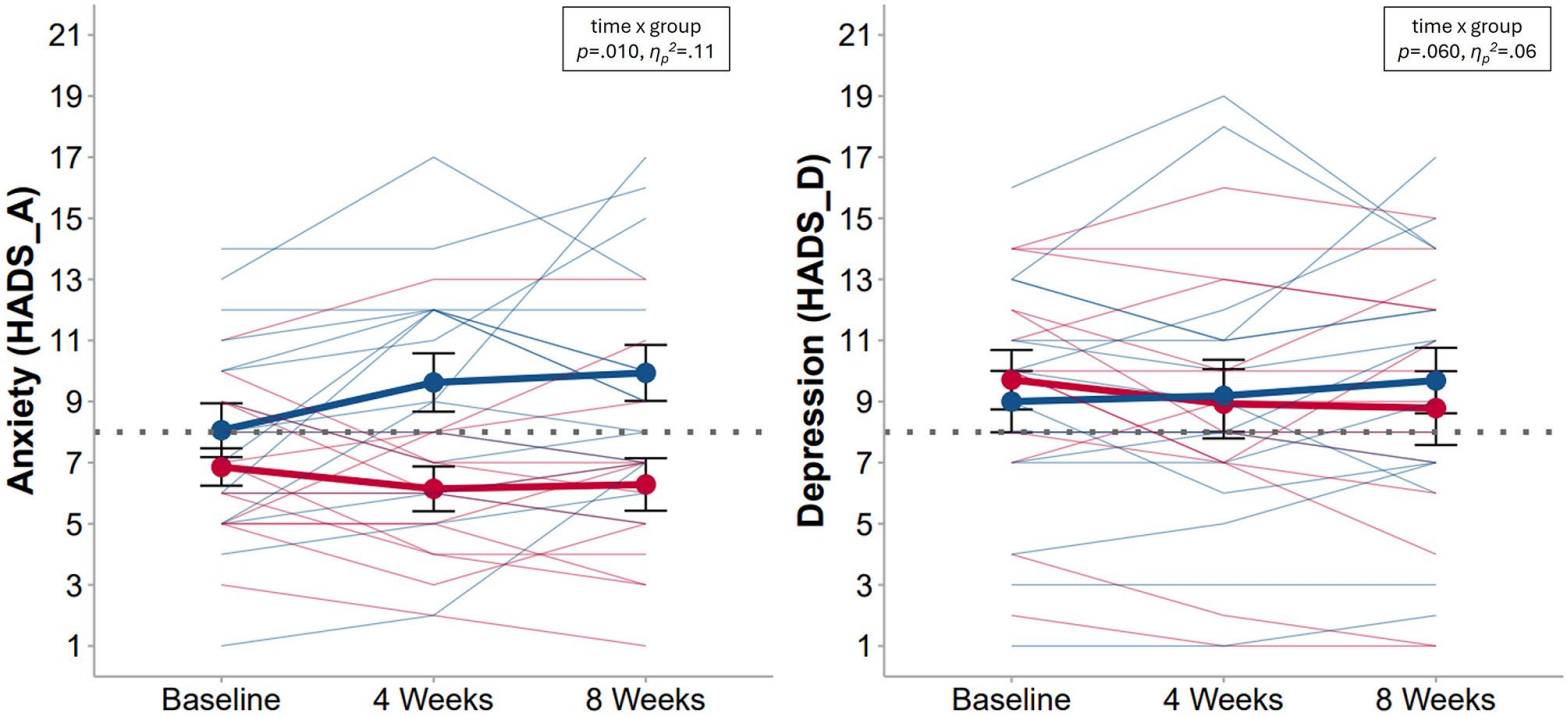
Group comparison for anxiety and depressive symptoms. *Note.* Analysed data from *n* = 30 participants who concluded the intervention period, of whom *n* = 14 in intervention group. Red lines = intervention group, blue lines = waitlist control group. Thin lines display individual trajectories, thick lines display group means (± standard errors), dotted lines represent the cut-off for subclinical symptoms. Subscales both range from 0–21. At baseline, *n* = 5 participants, of whom *n* = 2 in the intervention group, reported scores below eight (i.e., cut-off for subclinical symptoms) on both subscales. HADS = Hospital Anxiety and Depression Scale

Table 3 shows means and standard deviations for both groups and interference tests for primary and secondary outcome variables from linear mixed models across the 8-week intervention period. Anxiety symptoms decreased significantly more over 8 weeks in the intervention group than in the control group, but there was no significant time x group interaction for depressive symptoms (see time x group interaction for HADS-A and HADS-D in Table 3). Figure 4 displays these changes. Sensitivity analyses (see Suppl. 7) indicate the robustness of these results on anxiety. However, when outliers for HADS-D are removed, the time x group interaction becomes significant.

**Table 3:** Means, standard deviations, and fixed effects using linear mixed models

	Intervention		Control		Linear Mixed Models				
	M	SD	M	SD	Effect	df	F	*p*	η_*p*_^2^
Primary Outcomes									
Anxiety (HADS-A)									
Baseline	6.86	2.28	8.06	3.53	Time	58.000	2.02	0.161	0.03
4 Weeks	6.14	2.74	9.63	3.83	Group	66.321	0.06	0.815	0.00
8 Weeks	6.29	3.22	9.94	3.66	Group: Time	58.000	7.11	**0.010**	0.11
Depression (HADS-D)									
Baseline	9.71	3.65	9.00	4.02	Time	58.000	0.08	0.774	0.00
4 Weeks	8.93	4.25	9.19	4.72	Group	46.433	0.74	0.393	0.02
8 Weeks	8.79	4.53	9.69	4.29	Group: Time	58.000	3.73	0.060	0.06
**Secondary Outcomes**									
Self-reported stress (PSS-10)									
Baseline (*n* = 27)	17.17	6.64	17.87	6.81	Time	55.453	0.52	0.475	0.01
4 Weeks	16.57	5.98	19.81	7.44	Group	67.511	0.01	0.935	0.00
8 Weeks	15.86	6.06	20.31	5.61	Group: Time	55.453	3.10	0.084	0.05
Physical health impairment (CAT)									
Baseline	21.71	7.42	22.94	8.64	Time	58.000	1.88	0.175	0.03
4 Weeks	21.64	7.76	23.81	7.55	Group	42.396	0.12	0.728	0.00
8 Weeks	20.43	7.26	22.38	7.25	Group: Time	58.000	0.29	0.593	0.00
Dyspnoea (CRQ-SAS)									
Baseline	3.21	1.15	4.18	1.42	Time	58.000	1.72	0.194	0.03
4 Weeks	3.27	0.99	3.74	1.37	Group	56.190	2.49	0.120	0.04
8 Weeks	3.03	1.14	3.97	1.24	Group: Time	58.000	0.00	0.945	0.00
Fatigue (CRQ-SAS)									
Baseline	3.50	1.15	3.58	0.93	Time	58.000	0.41	0.523	0.01
4 Weeks	3.75	1.22	3.31	1.46	Group	50.940	0.14	0.709	0.00
8 Weeks	3.63	1.16	3.28	1.37	Group: Time	58.000	2.49	0.120	0.04
Emotional functioning (CRQ-SAS)									
Baseline	3.95	0.96	4.04	1.11	Time	58.000	0.01	0.939	0.00
4 Weeks	4.24	1.22	3.83	1.25	Group	46.648	0.68	0.415	0.01
8 Weeks	4.31	1.34	3.70	1.11	Group: Time	58.000	9.11	**0.004**	0.14
Mastery (CRQ-SAS)									
Baseline	3.79	1.24	3.97	1.15	Time	58.000	0.24	0.626	0.00
4 Weeks	4.28	1.41	3.94	1.49	Group	66.034	0.27	0.604	0.00
8 Weeks	4.09	1.34	3.84	1.19	Group: Time	58.000	1.39	0.244	0.02
Hair cortisol concentration (pg/mg)									
					Medication	23.400	3.93	0.059	0.14
Baseline (*n* = 23)	5.15	3.26	5.31	3.57	Time	20.489	0.12	0.734	0.01
4 Weeks					Group	23.836	0.22	0.646	0.01
8 Weeks (*n* = 25)	5.57	2.62	5.67	3.82	Group: Time	20.476	0.07	0.793	0.00
Stress reactivity - momentary subjective stress (VAS)									
Baseline	7.21	10.97	7.28	12.54	Time	51.238	10.33	**0.002**	0.17
4 Weeks (*n* = 25)	3.58	8.39	4.62	11.48	Group	76.931	0.08	0.777	0.00
8 Weeks (*n* = 27)	-0.15	6.31	2.79	12.06	Group: Time	51.238	0.42	0.521	0.01
Stress reactivity - HR (bpm)									
					Medication	25.741	0.04	0.838	0.00
Baseline (*n* = 28)	4.18	3.35	5.58	3.66	Time	26.483	0.05	0.832	0.00
4 Weeks (*n* = 26)	0.76	0.91	4.35	3.19	Group	26.821	0.10	0.754	0.00
8 Weeks (*n* = 26)	2.85	3.23	6.59	9.29	Group: Time	26.465	0.42	0.524	0.02
Stress reactivity - RMSSD (ms)									
					Medication	23.680	1.41	0.247	0.06
Baseline (*n* = 24)	0.39	6.68	0.94	10.81	Time	44.029	0.58	0.450	0.01
4 Weeks (*n* = 24)	0.93	8.65	1.77	13.98	Group	65.457	0.08	0.779	0.00
8 Weeks (*n* = 23)	3.45	8.94	1.13	4.00	Group: Time	44.106	0.00	0.967	0.00
Stress reactivity - SCL (µS)									
					Medication	26.110	3.23	0.084	0.11
Baseline	2.68	2.29	4.22	2.27	Time	54.775	0.76	0.387	0.01
4 Weeks (*n* = 28)	2.35	2.25	3.34	2.09	Group	78.572	3.72	0.058	0.05
8 Weeks (*n* = 27)	2.58	2.31	3.32	2.17	Group: Time	54.789	0.46	0.501	0.01

Note. Analysed data from *n* = 30 who concluded the intervention period, of whom *n* = 14 in the intervention group, unless otherwise indicated. Linear mixed models represent the models with the best model fit for each outcome variable. Model for heart rate includes a random slope for time in addition to the random intercept for participants, while all other models only include a random intercept for participant. Medication represents the number of medications, added as grand-mean centred covariate to the models with biological outcome variables. Intraclass coefficients (ICC) for the null models were as follows: HADS-A ICC = 0.722, HADS-D ICC = 0.847, PSS-10 ICC = 0.721, CAT ICC = 0.883, CRQ-SAS dyspnoea ICC = 0.802, CRQ-SAS emotional functioning ICC = 0.837, CRQ-SAS fatigue ICC = 0.817, CRQ-SAS mastery ICC = 0.706, hair cortisol ICC = 0.740, stress reactivity momentary subjective stress ICC = 0.442, HR ICC = 0.130, RMSSD ICC = 0.543, and SCL ICC = 0.546. HADS = Hospital Anxiety and Depression Scale, PSS-10 = Perceived Stress Scale, CAT = COPD Assessment Test, CRQ-SAS = Chronic Respiratory Questionnaire Self-Administered Standardized, HR = heart rate, RMSSD = root mean square of successive difference, SCL = skin conductance level

### Secondary outcomes

While emotional functioning improved more over 8 weeks in the intervention group than in the control group, there were no statistically significant time x group interactions that would indicate an intervention effect for the PSS-10, CAT, other CRQ-SAS subscales, or hair cortisol (see Table 3) after 8 weeks. For stress reactivity, a paired t-test comparing the initial resting state and peak stress exposure due to the Stroop task for momentary subjective stress, HR, RMSSD, and SCL showed that the Stroop task induced stress at the baseline measurement for momentary subjective stress, HR, and SCL (all *p* <.005), but not for RMSSD (*t*(23) = -0.381, *p* =.353). Linear mixed models revealed no significant differences in stress reactivity (i.e., difference score between initial resting state and peak stress exposure) over 8 weeks between groups (see Table 3). Sensitivity analyses (see Suppl. 7) indicate the robustness of these results, except for the PSS-10 and CRQ fatigue, where the time x group interaction is significant when removing outliers.

### Exploratory outcomes

Table 4 shows means, standard deviations, and interference tests for momentary subjective stress, anxiety, and dyspnoea levels using the Borg scale immediately before and after the logged meditations across the intervention period. All three variables reduced significantly from pre- to post-meditation (see Table 4). Sensitivity analyses (see Suppl. 7) indicate the robustness of these results.

**Table 4:** Means, standard deviations, and pre-post meditation effects using linear mixed models

			Linear Mixed Models			
	M	SD	df	F	*p*	η_*p*_^2^
Stress (VAS)						
Pre	19.61	14.38	12.804	38.81	< 0.001	0.75
Post	13.28	11.16				
Anxiety (adapted HADS item)						
Pre	0.41	0.59	12.529	6.79	0.022	0.35
Post	0.32	0.49				
Dyspnoea (Borg Scale)						
Pre	2.97	1.54	12.943	30.59	< 0.001	0.70
Post	2.07	1.51				

Note. Analysed data from *n* = 14 intervention group participants who concluded the intervention period. Analysed data include *n* = 545 pre and *n* = 535 post data entries from completed (≥ 10 min) meditations. Intraclass coefficients (ICC) for the null models were as follows: momentary subjective stress ICC = 0.655, anxiety ICC = 0.613, dyspnoea ICC = 0.686. VAS = visual analogue scale, HADS = Hospital Anxiety and Depression Scale

## Discussion

This pilot randomised controlled trial investigated the feasibility and preliminary effects of a self-administered digital MBI in COPD patients with elevated anxiety and/or depression. Our results indicate the intervention’s feasibility and preliminary effects indicate improvements in anxiety symptoms and emotional functioning after 8 weeks, as well as reductions in momentary subjective stress, anxiety, and dyspnoea immediately after mindfulness meditation in everyday life.

### Primary outcomes

The results demonstrated fewer dropouts and higher usage rates compared to other MBI studies in COPD [13], which may be attributed to the digital intervention format [39], the brief meditations [40], and the inclusive assessment design in participants’ homes. Overall, participants reported positive experiences with the intervention and individual meditations, aligning with findings from similar studies [13]. However, long-term treatment adherence during the follow-up period was lower than participants intended, potentially influenced by absence of daily meditation reminders and weekly check-in calls, which might have facilitated practice [19]. Digital MBIs may therefore benefit from incorporating practice reminders and support mechanisms, possibly including messaging tools or peer support [39]. Researchers and clinicians may also consider offering greater variety in meditation types and/or lengths to promote personalisation and intervention-tailoring, and implement gamification and individual feedback to help participants sustain their practice [19, 41].

In line with our primary hypothesis and existing MBI literature [15, 18], we found a preliminary intervention effect on anxiety symptoms after 8 weeks. This represents a new and promising result for COPD research and clinical practice, warranting further investigation in large-scale studies. The results do not suggest a reduction in depressive symptoms after 8 weeks, which is contrary to our hypothesis as well as the MBI literature [15, 18] and findings from mindfulness-based cognitive therapy (MBCT) in COPD [27]. MBCT in its original form was designed for patients with depressive symptoms, and the cognitive-behavioural elements may especially target depressive symptoms. However, the differing effects may also be explained by different delivery formats, the combination of the MBCT with pulmonary rehabilitation, or other methodological differences, as another tele-delivered MBCT in COPD likewise reported no significant effects on depression [39]. Importantly, given the small sample size and the preliminary nature of the intervention effects, these results should not be overinterpreted and should be further investigated in larger trials.

### Secondary outcomes

The preliminary intervention effect suggesting an improvement in emotional functioning after 8 weeks relates closely to the primary outcomes, further supporting the finding that MBIs may improve psychological symptoms in COPD, especially on an affective level. Contrary to our hypothesis, we did not find any intervention effects on physical health impairment or COPD-related quality of life after 8 weeks, consistent with previous studies [13]. However, the effect sizes of these results were very small to small and need to be interpreted with caution, warranting further exploration in future studies.

Contrary to our hypothesis but in line with a previous study [42], we did not find preliminary intervention effects on self-reported stress after 8 weeks, although this was not robust in the sensitivity analysis. Patients reported elevated baseline levels of self-reported stress [28], indicating a high risk of chronic stress. Given the potential clinical relevance, along with the lack of evidence and promising trends for stress reduction identified in this study, future large-scale studies should investigate the role of stress in COPD, and potential effects of MBIs.

Furthermore, we found no preliminary intervention effects indicating changes in the functioning of the assessed bodily stress systems. Specifically, we found no effects on hair cortisol (representing basal HPA axis activity) or stress reactivity to a cognitive stressor (assessing heart rate, heart rate variability, and skin conductance level as correlates of ANS reactivity) after 8 weeks. It is possible that the administered cognitive stressor did not induce sufficient stress levels to detect ANS regulatory functions, or that habituation to the stressor may have been too strong to detect an intervention effect. The administered MBI may not have been sufficiently intense to induce changes in basal HPA axis activity or ANS reactivity (e.g., cf [16]). Further investigation into stress system activity and reactivity in COPD patients is necessary to understand how MBIs might effectively target biological stress systems in this group.

### Exploratory outcomes

Our findings indicate a reduction in momentary subjective stress, anxiety, and dyspnoea immediately following self-administered meditations, suggesting that brief meditations may elicit short-term benefits in everyday life and hold promise for clinical applications and COPD outpatient care. Similar effects were observed in a study investigating a 20-minute mindful breathing meditation for dyspnoea [43], whereas another study exploring a brief body scan reported no significant immediate effects [40]. While our results imply short-term reductions for dyspnoea, the sustainability of the investigated MBI appears limited, indicated by the lacking effects on CRQ dyspnoea and CAT. Notably, participants mainly meditated during moments of low stress, anxiety, and dyspnoea. While this highlights the intervention’s potential in relaxed situations, the feasibility and effectiveness in situations of high stress, anxiety, and dyspnoea need to be explored in future studies.

### Strengths and limitations

The study strengths include the randomised design, follow-up period, biomarker inclusion, qualitative results supplementing questionnaire data, a well-characterised sample selection, and the administration of an adapted intervention for COPD patients. However, some limitations should be acknowledged, including the small sample size. Consequently, the intervention effects are preliminary and should be viewed as trends that require further exploration in future large-scale studies. Additionally, the use of a passive control group and the absence of a control condition for exploratory analysis (as this was a pre-post meditation comparison within the intervention group) should be noted as limitations regarding the intervention effects. Generalisability to all COPD patients is limited, as a considerable number of individuals were either ineligible or not interested in study participation. Self-selection of participants may have led to a higher percentage of prior experiences with psychological professionals (65.8%) and mind-body interventions (65.8%) compared to the general COPD population. However, as patients reported that these experiences primarily occurred during pulmonary rehabilitation—a standard treatment available to COPD patients in Austria, which includes psychological consultation and typically also relaxation training—the percentages may be representative of the general Austrian COPD population. Recruitment from institutions where patients already showed treatment interest may have influenced participant adherence. Additionally, providing waitlist participants with the intervention following the intervention period hindered a clear group comparison at follow-up.

## Conclusions

The investigated self-administered digital MBI, including brief 10-15-minute meditations, was feasible and holds potential as low-threshold add-on treatment to alleviate anxiety after 8 weeks and reduce momentary subjective stress, anxiety, and dyspnoea in everyday life. Digital MBIs for COPD patients may offer a variety of meditations, feature reminders, and support mechanisms. Future studies should investigate digital MBIs in large-scale trials with active control conditions, follow-up periods, and assessments in everyday life to confirm the preliminary intervention effects observed in this study. Researchers should further explore the potential of MBIs in moments of acute breathlessness to help manage exacerbations in daily life. This may ultimately lead to the implementation of digital MBIs as flexible, personalised, easy-to-administer, and cost-effective add-on treatments in clinical settings and patients’ everyday lives.

## Electronic supplementary material

Below is the link to the electronic supplementary material.

Supplementary Material 1

## Data Availability

De-identified data from this study are available (as permitted according to the standards of the ethics review board of the city of Vienna) by contacting the corresponding author on reasonable request. Analytic code will be made available by contacting the corresponding author. Study materials are available as digital supplements and on OSF (https://osf.io/sx5h9/). Further materials will be made available by contacting the corresponding author.

## Footnotes

1 The use of the Chronic Respiratory Questionnaire Self-Administered Standardized, authored by Guyatt and Schünemann, has been allowed under license from McMaster University, Hamilton, Canada.

## Abbreviations

ANOVA: Analysis of variance
ANS: Autonomic nervous system
CAT: COPD assessment test
COPD: Chronic obstructive pulmonary disease
CRQ-SAS: Chronic Respiratory Questionnaire Self-Administered Standardized
HADS-A: Hospital Anxiety and Depression Scale-Anxiety
HADS-D: Hospital Anxiety and Depression Scale-Depression
HPA: Hypothalamic-pituitary-adrenal (axis)
HR: Heart rate
ICS: Inhaled corticosteroid
MBCT: Mindfulness-based cognitive therapy
MBI: Mindfulness-based intervention
PSS-10: Perceived Stress Scale (10-item version)
RMSSD: Root mean square of successive differences
SCL: Skin conductance level
TAU: Treatment as usual
