# Supplementary material for: Digital mindfulness-based intervention for people with COPD – a multicentre pilot and feasibility RCT
Source: Respir Res. 2025 May 26;26:199. doi: 10.1186/s12931-025-03243-4 (PMC12105243; doi:10.1186/s12931-025-03243-4)
Supplement: Supplementary file 1 — Supplementary Material 1 [file 12931_2025_3243_MOESM1_ESM.docx]

**ONLINE SUPPLEMENTS**

# Supplementary Methods

## Suppl 1. *Covid-19 Protocol*

During the entire study period, a strict COVID-19 protocol was administered to ensure safe in-person contact with participants, which had been adjusted in line with changing COVID-19 regulations in Austria over time. Assessors were vaccinated with a COVID-19 vaccine, provided a negative SARS-CoV-2 antigen test result (on the day of the assessment) or a polymerase chain reaction test result (conducted no more than 48 hours before the assessment), were free of any symptoms of illness, wore an FFP2 face mask during the entire assessment as well as gloves for any skin contact with patients (e.g. when collecting hair samples), followed general hygiene rules (e.g. hand disinfection), and disinfected all equipment before and after each assessment. Patients did not wear an FFP2 face mask or were tested for COVID-19, but assessors checked for any symptoms of illness in a telephone call one day prior to the assessment and postponed or cancelled the assessment if any symptoms were present (with the exception of their regular COPD symptoms). The assessments were never paused during the study period, as professional in-person visits were always allowed during lockdowns in Austria.

## Suppl 2. *Stress Induction Protocol*

The stress induction protocol used in this study was a modified Stroop task (inspired by [1, 2]), consisting of 7 cards with 45 words (coloured in brown, green, red, blue, and yellow) presented in three columns with 15 words each. Words and colours were ordered semi-randomly (no same words/colours in a row, each column contains each word/colour at least once). The cards presented positive words (1x) negative words (1x), COPD-related somatic words (1x) matched neutral words (2x), and colour words (2x). Positive (e.g., perfect), negative (e.g., lie), colour, and neutral (e.g., chair) words originated from [1–5]. COPD-related somatic words (e.g., chough) were extracted from interview transcripts with COPD patients [6], depending on their frequency and relevance for the interviewed patients. A list of 37 extracted words was rated by three experts (pulmonologists) regarding their relevance for COPD patients on a five-staged scale ranging from 0 = “not relevant at all” to 4 = “very relevant”. Mean ratings were calculated for each word, and the ten most relevant words were chosen for the task. All word cards were matched in terms of character length and frequency of use in the German language. The words were printed on white A4 size cards.


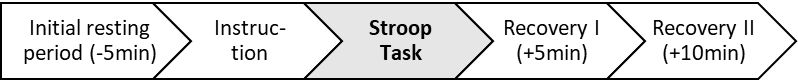

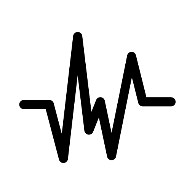

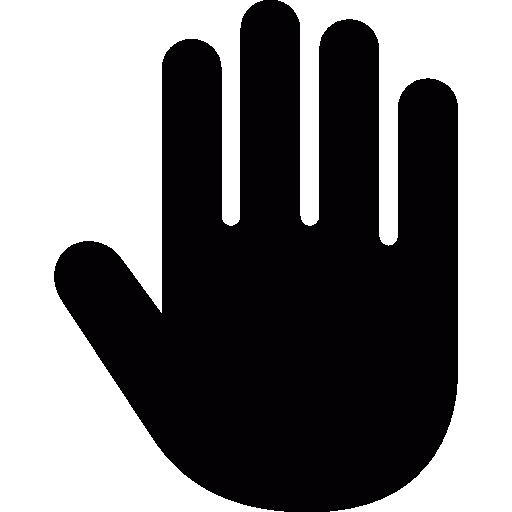

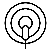

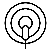


ongoing measurement of autonomic nervous system markers


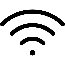

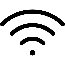

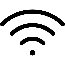

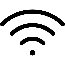

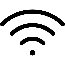

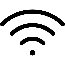

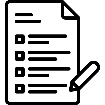

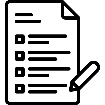

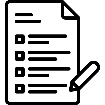

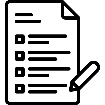

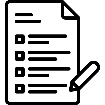


**Figure S1.**

*Procedure of stress induction protocol.* Equipment for the measurement of the autonomic nervous system activity (skin conductance level (SCL) using palm electrodes and an EDA sensor, heart rate using a chest belt with electrocardiogram ECG electrodes and an ECG sensor) was attached before the stress induction protocol. Markers (signal icon) were set six times on the EDA sensor. Patients reported momentary subjective stress, dyspnea, anxiety, and relaxation (chart icon) five times. Icons provided by [www.flaticon.com](http://www.flaticon.com/).

At the beginning of each assessment, assessors attached the equipment to monitor ANS activity (for details see Supplement 5*)* to ensure an ongoing and stable measurement of ANS activity during the stress induction protocol (initial resting period, Stroop task, recovery). Figure S1 depicts the procedure of the stress induction protocol. The Stroop task was preceded by a 5 min initial resting period and followed by a 2x5 min resting period (recovery I, recovery II), in which participants were instructed to sit upright and quietly on a chair. They could read a magazine and ask questions, but interactions were not encouraged during resting periods. After the initial resting period, participants were instructed on the procedure of the Stroop task. Participants should state the printed colour instead of the word itself as fast and as error-free as possible, which requires selective attention and cognitive inhibition of the presented words. After a control card presenting coloured Os (random number of Os varying between 4, 5, 6, 7, 9, 11; number matched to the character length of the used words), the 7 word cards were presented in a random order. The assessor noted reaction time (using a stopwatch) and mistakes for each card. Further, the task was audio-taped (using the voice recorder app on the assessors’ smartphone) to subsequently check the noted times and mistakes. The Stroop task lasted between 3 and 15 min (termination by the assessors if the Stroop lasted longer than 15 min), while the introduction lasted between 2 and 8 min. On five measurement time points (5min before Stroop, before Stroop, after Stroop, 5min after Stroop, 10min after Stroop), patients reported their momentary subjective stress (visual analogue scale, ranging from 0 to 100, printed on paper as a 100mm line where participants indicated their stress level with a cross on that line), dyspnoea (modified Borg Scale, ranging from 0 “no shortness of breath at all” to 10 “maximal shortness of breath”, [7, 8]), anxiety (items from Hospital Anxiety and Depression Scale, ranging from 0 “not at all” to 3 “very”, [9]), and relaxation (item from Multidimensional Mood State Questionnaire, ranging from 1 “not at all” to 5 “very”, [10]). Additionally, the assessor set a marker on the EDAMove4 sensor (Movisens GmbH) positioned on the non-dominant hand of the participant in order to be able to identify the different periods of the stress induction protocol in the ANS data (initial resting period, introduction, Stroop task, recovery I, recovery II). After the recovery phase, participants took off the equipment to monitor ANS activity.

## Suppl 3. *Telephone Interview*

A telephone interview was conducted a few days after T_2_ by the study coordinator, who was experienced in conducting interviews. The interviews lasted between 15 and 47 minutes (duration (min): *M* = 31, *SD* = 11). They were audio-recorded using the app Samsung Voice Recorder (Version 21.4.00.28; Samsung Electronics Co.) on a smartphone (Samsung A12). The interviewer took notes about the patient's answers as well as relevant interview and patient characteristics. The interview questions were inspired by previous studies assessing feasibility in interviews [11] and adapted by the authors. The interview covered an open-ended entry question, closed questions about the study design as well as closed questions with open-ended follow-up questions covering participant's practice behaviour, ease of use and integration into everyday life, hindering and motivating factors, experienced effects, and satisfaction with the intervention, where participants could elaborate in more detail. In addition, participants rated how much they liked the single exercises of the intervention on a 5-staged Likert scale from 0-4 (“not at all” to “very much”) and if they would recommend the exercises to other patients with COPD (dichotomous item, yes/no). The answers to all questions have been noted by the interviewer, verified using the audiotapes, and have been analysed descriptively. Additionally, answers to the open-ended questions have been analysed in more detail using the thematic analysis [12].

## Suppl 4. *Post-monitoring Questionnaire*

The post-monitoring questionnaire was conducted as a structured interview at the end of T_4_ by the assessors, who noted the answers on the questionnaire. In addition to questions addressing the study design (e.g., “I often found the hair sampling bothering”) or the study period in general (e.g., “The 6 months of study participation reflect my usual life situation”), which served as control questions and informed an adequate interpretation of the data as well as the planning of future studies with COPD patients, three dichotomous items assessed the long-term adherence to the self-administered mindfulness practice in the follow-up period, namely “Did you conduct mindfulness activities during the last 4 months?”, “Did you use the study mindfulness activities during the last 4 months?”, “During the past 4 months, have you performed other mind-body techniques such as relaxation exercises, meditation, autogenic training, imaginative processes, yoga, qi gong, or tai chi?”. The answers to all questions have been noted by the interviewer and have been analysed descriptively.

## Suppl 5. *Assessment of Biomarkers*

**Heart rate and heart rate variability.** Heart rate and heart rate variability were assessed using the EcgMove4 sensor (ECG 1024 Hz, Movisens GmbH), which was attached to a chest belt with two embedded dry electrodes. The belt was positioned directly under the sternum. The software DataAnalyzer (Movisens GmbH) converted the electrocardiogram signals into heart rate (HR) (bpm) and root mean square of successive difference (RMSSD) (ms).

**Skin conductance level.** Skin conductance level (SCL) was assessed using the EdaMove4 sensor (Movisens GmbH) with a sample rate of 32 Hz, which is attached to a wrist band with two solid gel silver chloride electrodes. The sensor uses the exosomatic measurement method, applying a constant DC voltage of 0.5 V. The electrodes are attached to the palm of the non-dominant hand using adhesive tape. The software DataAnalyzer (Movisens GmbH) converted the electric signal into SCL (µS).

**Hair cortisol.** For the hair sampling, 2 to 4 small hair strands were cut from the posterior vertex region (back head) of the scalp. Hair strands were cut as closely as possible to the scalp, as the scalp-near 2 cm segments indicated the cumulative hair cortisol concentration of the last 2 months. The samples were stored in a dry place at room temperature, until they were analysed in the Biochemical laboratory of the Faculty of Psychology, University of Vienna.

## Suppl 6. *Data Analysis and Data Preprocessing*

**Primary outcomes**

*Dropout rate.* We utilized the assessment protocols to determine the number of participants completing each measurement (Baseline, 4 weeks, 8 weeks) and subsequently calculated the dropout rate for the intervention period.

*Usage behaviour.* To analyse usage behaviour, we employed participant log data obtained from the MovisensXS (Movisens GmbH) software. Initially, we exported the data file from the software and manually incorporated information from a participant who encountered a technical issue resulting in data loss. Despite losing their pre and post responses (momentary subjective stress, anxiety, dyspnea), we were able to supplement the usage data using their handwritten notes. Dummy variables were generated for day (0 – 56), prepost (0 = pre, 1 = post meditation), missing (0 = not missing, 1 = missing data entry), and for each meditation (1 = conducted meditation, 0 = did not conduct meditation). Additionally, meditation length was converted from ms into min (time from starting the audio file until the participant proceeds to the post questions). Another dummy variable, executed, was created to indicate missing meditations, incomplete meditations (<10 minutes), and complete meditations (≥10 minutes). Test data entries from the face-to-face introduction were removed, as well as entries beyond day 56 if the 8-week measurement could not be conducted exactly after day 56. Finally, the file was reviewed for unintentionally triggered data entries or software errors. For the calculation of usage data, only the subset of data with completed meditations was used.

*Telephone interview.* The assessors' noted responses were digitized, cross-checked with the audio transcripts, and subsequently analysed using descriptive statistics. Due to technical issues, one audio recording could not be transcribed. Additionally, another audio file exhibited very low quality, rendering parts of the participant's responses incomprehensible. The thirteen audio files were fully and verbally transcribed by two research assistants and then imported into the MAXQDA software. Subsequently, they underwent coding and structuring using thematic maps, following an iterative process based on the steps recommended for thematic analysis [12].

*Post-monitoring questionnaire:* We used the noted responses from the assessors, digitized them, verified the accuracy of the digital data transfer, and calculated frequencies.

*Symptoms of anxiety and depression.* The described procedure applies to all questionnaire data, including secondary outcomes and baseline measures. We utilized data from the questionnaires collected during the measurements, digitalized them, and reviewed the digital data for accuracy. For analysis, we exclusively considered data from baseline, 4 weeks, and 8 weeks measurements, as these measurements allow for a clear interpretation of the intervention effects in comparison to the passive waitlist control group. Subsequently, we calculated individual scales and utilized both sum and mean scores for further analyses. No corrections were applied for multiple testing since we did not employ any items for multiple hypothesis testing or test one hypothesis with multiple statistical tests [13]. We interpret subscales of questionnaires based on what the subscale measures (e.g., HADS-A for anxiety, HADS-D for depression), not the entire scale (e.g., HADS for psychological distress). Moreover, we did not impute any missing data, as linear mixed models can handle missing data points [14]. Outliers were not excluded. Nonetheless, sensitivity analyses were conducted without outliers. For the fitting of our linear mixed models, we utilized the R packages lme4 [15], performance [16], and effectsize [17]. Restricted likelihood estimation and the Kenward-Roger approximation were employed for estimating effective degrees of freedom in small samples [18]. The null model was compared with models including relevant fixed and random effects, and the model with the best fit for each outcome variable was selected. Visual inspection of model assumptions was carried out using the check_model function from the performance package. Linear mixed models are relatively robust against deviations from model assumptions [19]. Therefore, we chose not to alter the data by using transformations or other attempts to normalize the distribution of residuals or random effects, nor by excluding outliers. However, sensitivity analyses were conducted, removing outliers and employing the robustlmm R package [20] for a robustness check on our analysis. Effect size (partial eta squared) was calculated using the F values from the chosen model. Finally, two-sample t-tests were conducted to compare the groups at each measurement (baseline, 4 weeks, 8 weeks) individually.

**Secondary outcomes**

*Questionnaires.* For the questionnaire data, see the section above.

*Hair cortisol:* For the analysis, 2.0 ± 0.2 cm segments of scalp-near hair were examined in the biochemical laboratory at the Faculty of Psychology, University of Vienna. The laboratory protocol was based on Stalder et al. [21] with slight modifications. Hair samples underwent a dual 3-minute washing using 3 mL of isopropanol. For cortisol extraction, 10 ± 0.5 mg of cut hair was incubated in 1.8 mL of methanol for 18 hours at room temperature. Following incubation, 1.6 mL was transferred to another glass vial, and the supernatant underwent evaporation at 50°C until complete drying of the samples. Subsequently, the dried samples were resuspended in 225 µL of HPLC ultra-pure water and vortexed for 20 seconds. Cortisol determination utilized a commercially available cortisol luminescence immunoassay (LIA; IBL International, a Tecan Group company, Hamburg, Germany). Both inter- and intra-assay coefficients of variation were below 10% for all assays. Hair cortisol levels were then correlated with possible influencing variables (sex, age, BMI, hair washing frequency, cosmetic hair treatment, heat treatment, number of medications, and all specified medications: LAMA, LABA, ICS, Roflumilast, Bronchodilator, other). Pearson’s product-moment correlation was significant for the number of medications, ICS, and Roflumilast. As ICS and Roflumilast are already represented in the number of medications, we decided to include only this covariate in the models. However, we conducted sensitivity analyses including those covariates as well. The number of medications was included in the models as a level 2 covariate, which was centred at the grand mean using the R package misty (Yanagida, 2024, version 0.6.2). Hair cortisol concentration was analyzed analogously to the questionnaire data, except for applying three steps in the model fitting, starting with the null model, followed by the model including the control variable, and, finally, the model including all fixed effects (predictors and control variable) [14]. There were no outliers for hair cortisol concentration.

*Stress Reactivity:* For subjective stress, we utilized the data provided by participants during the stress induction protocol at different measurements. Using a ruler, we identified participants' marked responses on the 100mm line (visual analogue scale from 0 to 100), where mm represent the values on the visual analogue scale (VAS). These values were digitized and reviewed to ensure accurate data transfer. Data for HR, RMSSD, and SCL were collected via Movisens (Movisens GmbH) electrodes (see Suppl. 5). Electrocardiogram recordings displaying multiple artifacts were excluded from further processing. Electric and electrocardiogram signals were collected throughout the entire measurement and needed to be segmented into snippets of data for the five phases of the Stroop task (see Suppl. 2). This segmentation was achieved by using markers set on the electrodes by the assessors, representing specific time points in the electrocardiogram/electric signal recording. The initial resting period, recovery I, and recovery II were each cut into 5-minute snippets, while the other two phases had variable lengths depending on the Stroop task duration. In case of missing markers, the missing time points were identified using the audio recordings of the Stroop task and manually inserted into the electrocardiogram/electric signal recordings. SCL data were segmented manually, while HR data were segmented using a Python code specifically written for this purpose (contact authors for further details). Using the DataAnalyzer software (Movisens GmbH), we converted the snippets of the electrocardiogram/electric signal recordings into heart rate (HR) (bpm), root mean square of successive difference (RMSSD) (ms), and SCL (µS). We employed 1-minute intervals, meaning that for each 1-minute interval of the recording, an HR, RMSSD, or SCL data value was extracted. The individual values were then merged, and mean values of the 1-minute intervals were calculated for each phase of the stress induction protocol. For RMSSD, the program could not convert data for all phases, resulting in program-based missing values. No outliers were excluded for HR and SCL data, as all outliers were within participants with generally higher/lower HR and SCL values, respectively. For RMSSD data, eight outliers were excluded as they were likely produced by missing values and technical issues. No other outliers were excluded.

The final data sheet included the variables: participant ID for the 30 analysed participants, group (1 = intervention, 2 = waitlist), day (1 = baseline, 2 = 4 weeks, 3 = 8 weeks, 4 = 4 months, 5 = 6 months), and the time points within the stress induction protocol (for VAS: 1 = 5min before Stroop, 2 = before Stroop, 3 = after Stroop, 4 = 5min after Stroop, 5 = 10min after Stroop; for mean values HR, mean values RMDDS, and mean values SCL: 1 = initial resting period, 2 = instruction, 3 = Stroop task, 4 = recovery I, 5 = recovery II). Including only days 1, 2, and 3 for the 30 analysed participants (450 data points), we had 424 data points for VAS (5.78% missing), 428 for SCL (4.89% missing), 404 for HR (10.22% missing), and 361 for RMSSD (19.78% missing). To determine if the Stroop was able to induce stress at baseline, we used the subset of the data with day = 1 and compared time point 1 and time point 3 for each variable using paired t-tests in SPSS. We then calculated stress reactivity as the difference score between time point 3 and 1 for each day and participant. For the difference scores, positive values represent an increase in stress from time point 1 to 3, while negative values represent a decrease from time point 1 to 3. For the group comparison over the intervention period, we used days 1-3 and the difference scores of each variable. Linear mixed models for subjective stress were fitted analogously to the procedure described for questionnaires, while linear mixed models for HR, RMSSD, and SCL were fitted similarly to hair cortisol concentration for better comparability. Correlation with other potentially influencing covariates was checked during sensitivity analyses.

**Exploratory outcomes**

For the exploratory outcomes, we utilized the same data file as for the usage data, employing a subset of data only containing completed meditations. No outliers were excluded. Regarding model fitting, we used the same approach as described for questionnaire data, with the only distinction being the presence of only one fixed effect, prepost (0 = pre, 1 = post-meditation).

## Suppl 7. *Sensitivity Analyses*

Sensitivity analyses included: 1) Removing outliers (considering grouping factors time and group), 2) Linear mixed models with the R package robustlmm to check robustness of results. The robust model was calculated using the function rlmer. The p-values of the robust model were calculated using the degrees of freedom from the no-robust model. 3) Including different covariates and removing the covariate number of medication from the analysis with biological outcomes.

**HADS-A:** 1) 1 outlier, rejection/acceptance of hypothesis did not change when removing outliers. 2) Rejection/acceptance of hypothesis did not change when fitting the robust model.

**HADS-D:** 1) 2 outliers, removing the outliers resulted in a significant time x group interaction (*p* = .043). 2) Rejection/acceptance of hypothesis did not change when fitting the robust model.

**PSS:** 1) 7 outliers, removing the outliers resulted in a significant (*p* = .0348) time x group interaction. 2) Rejection/acceptance of hypothesis did not change when fitting the robust model.

**CAT:** 1) 9 outliers, rejection/acceptance of hypothesis did not change when removing outliers. 2) Rejection/acceptance of hypothesis did not change when fitting the robust model.

**CRQ dyspnoea:** 1) 3 outliers, rejection/acceptance of hypothesis did not change when removing outliers. 2) Rejection/acceptance of hypothesis did not change when fitting the robust model.

**CRQ fatigue:** 1) 7 outliers, removing the outliers resulted in a significant (*p* = .007) time x group interaction. 2) Rejection/acceptance of hypothesis did not change when fitting the robust model.

**CRQ emotional functioning:** 1) 1 outlier, rejection/acceptance of hypothesis did not change when removing outliers. 2) Fitting of the robust model resulted in a significant main effect of time (*p* = .011) in addition to the significant interaction effect of time x group (*p* = .001), which was also found in the non-robust model.

**CRQ mastery:** 1) 2 outliers, rejection/acceptance of hypothesis did not change when removing outliers. 2) Rejection/acceptance of hypothesis did not change when fitting the robust model.

**Hair cortisol:** 1) 1 outlier, rejection/acceptance of hypothesis did not change when removing outliers. 2) Rejection/acceptance of hypothesis did not change when fitting the robust model. 3) Rejection/acceptance of hypothesis did not change when removing all covariates or adding the other grand-mean cantered level 2 covariates hair cortisol level correlated with (ICS, Roflumilast, ICS + Roflumilast).

**Stress reactivity – VAS:** 1) 6 outliers, rejection/acceptance of hypothesis did not change when removing outliers. 2) Rejection/acceptance of hypothesis did not change when fitting the robust model.

**Stress reactivity – HR:** 1) 1 outlier, removing the outliers resulted in a significant main effect of time (*p* = .038). 2) Rejection/acceptance of hypothesis did not change when fitting the robust model. 3) Rejection/acceptance of hypothesis did not change when removing all covariates or adding the other grand-mean cantered level 2 covariates hair cortisol level correlated with (Roflumilast).

**Stress reactivity – RMSSD:** 1) 11 outliers, rejection/acceptance of hypothesis did not change when removing outliers. 2) Rejection/acceptance of hypothesis did not change when fitting the robust model. 3) Rejection/acceptance of hypothesis did not change when removing all covariates or adding the other grand-mean cantered level 2 covariates hair cortisol level correlated with (LAMA, Roflumilast, LAMA + Roflumilast).

**Stress reactivity – SCL:** 1) 6 outliers, removing the outliers resulted in a significant main effect of time (*p* = .012) and group (*p* = .027). 2) Rejection/acceptance of hypothesis did not change when fitting the robust model. 3) Rejection/acceptance of hypothesis did not change when removing all covariates or adding the other grand-mean cantered level 2 covariates hair cortisol level correlated with (age, BMI, age + BMI).

**Momentary subjective stress:** 1) 8 outliers, rejection/acceptance of hypothesis did not change when removing outliers. 2) Rejection/acceptance of hypothesis did not change when fitting the robust model.

**Momentary anxiety:** 1) no outliers. 2) Rejection/acceptance of hypothesis did not change when fitting the robust model.

**Momentary dyspnoea:** 1) 2 outliers, rejection/acceptance of hypothesis did not change when removing outliers. 2) Rejection/acceptance of hypothesis did not change when fitting the robust model.

# Supplementary Tables

## **Table S1.** *Baseline characteristics*

|  | **Intervention**  **(*n* = 17)** | | **Waitlist**  **(*n* = 16)** | | **Total**  **(*n* = 38)** | |
| --- | --- | --- | --- | --- | --- | --- |
|  | ***n*** | **%** | ***n*** | **%** | ***n*** | **%** |
| Age (*M* ± *SD*; Range) | 62.59 ± 7.75;  47 - 77 | | 62.06 ± 5.93;  53 - 72 | | 62.68 ± 7.07;  47 - 77 | |
| Gender |  |  |  |  |  |  |
| Male | 8 | 47.1 | 5 | 31.3 | 15 | 39.5 |
| Female | 9 | 52.9 | 11 | 68.8 | 23 | 60.5 |
| BMI (*M* ± *SD*; Range) | 25.45 ± 5.97;  17 - 38 | | 24.06 ± 2.62;  19 - 28 | | 25.14 ± 4.75;  17 - 38 | |
| Duration COPD in years (*M* ± *SD*; Range) | 9.12 ± 5.28;  1 - 20 | | 10.47 ± 5.50;  5 - 23 | | 10.46 ± 6.13;  1 - 23 | |
| FEV1 Liter (M ± SD; Range) (*n = 36*) | 1.06 ± 0.67;  0 - 3 | | 1.14 ± 0.59;  1 - 2 | | 1.18 ± 0.67;  0 - 3 | |
| FEV1 Percent (M ± SD; Range) (*n = 36*) | 35.46 ± 16.29; 18 - 77 | | 41.8 ± 19.79;  20 - 82 | | 41.21 ± 19.16; 18 - 82 | |
| FEV1/FVC Percent (*M* ± *SD*; Range) (*n = 35*) | 45.44 ± 15.26; 30 - 85 | | 43.26 ± 13.16; 28 - 66 | | 46.99 ± 14.94; 28 - 85 | |
| COPD Stage *(n = 36)* |  |  |  |  |  |  |
| I | 1 | 5.9 | 0 | 0 | 1 | 2.8 |
| II | 3 | 17.6 | 1 | 6.7 | 4 | 11.1 |
| III | 2 | 11.8 | 9 | 60.0 | 14 | 38.9 |
| IV | 11 | 64.7 | 5 | 33.3 | 17 | 47.2 |
| Medical conditions |  |  |  |  |  |  |
| No other medical conditions | 8 | 47.1 | 8 | 50 | 17 | 44.7 |
| Pervious | 1 | 5.9 | 3 | 18.8 | 4 | 10.5 |
| Current (acute or chronic) | 8 | 47.1 | 5 | 31.3 | 17 | 44.7 |
| Medical conditions - Specification |  |  |  |  |  |  |
| Diabetes (acute or chronic) | 2 | 11.8 | 0 | 0 | 2 | 5.3 |
| Lung cancer (previous) | 1 | 5.9 | 1 | 6.3 | 2 | 5.3 |
| Coronary heart disease (previous) | 1 | 5.9 | 1 | 6.3 | 2 | 5.3 |
| Coronary heart disease (acute or chronic) | 1 | 5.9 | 0 | 0 | 2 | 5.3 |
| Osteoporosis (acute or chronic) | 1 | 5.9 | 4 | 25 | 6 | 15.8 |
| Other (previous) | 1 | 5.9 | 3 | 18.8 | 4 | 10.5 |
| Other (acute or chronic) | 8 | 47.1 | 2 | 12.5 | 13 | 34.2 |
| Psychiatric disorder |  |  |  |  |  |  |
| No | 13 | 76.5 | 11 | 68.8 | 27 | 71.1 |
| Pervious | 2 | 11.8 | 2 | 12.5 | 5 | 13.2 |
| Current (acute or chronic) | 2 | 11.8 | 3 | 18.8 | 6 | 15.8 |
| Psychiatric disorder - Specification |  |  |  |  |  |  |
| Depression/Burnout* (previous) | 2 | 11.8 | 2 | 12.5 | 4 | 10.5 |
| Depression (acute or chronic) | 0 | 0.0 | 3 | 18.8 | 4 | 10.5 |
| Anxiety/Panic disorder* (previous) | 1 | 5.9 | 0 | 0 | 1 | 2.6 |
| Anxiety disorder (acute or chronic) | 1 | 5.9 | 0 | 0 | 1 | 2.6 |
| Panic attacks* (acute or chronic) | 0 | 0 | 1 | 6.3 | 1 | 2.6 |
| Substance abuse | 1 | 5.9 | 0 | 0 | 1 | 2.6 |
| Number of prescribed medications  (*M* ± *SD*; Range) | 6.35 ± 2.57;  4 - 12 | | 5.25 ± 3.55;  0 - 12 | | 5.74 ± 3.12;  0 - 12 | |
| LAMA | 15 | 88.2 | 15 | 93.8 | 34 | 89.5 |
| LABA | 16 | 94.1 | 14 | 87.5 | 33 | 86.8 |
| ICS | 15 | 88.2 | 12 | 75 | 29 | 76.3 |
| Roflumilast | 2 | 11.8 | 3 | 18.8 | 5 | 13.2 |
| Bronchodilator | 13 | 76.5 | 10 | 62.5 | 25 | 65.8 |
| Other | 15 | 88.2 | 11 | 68.8 | 29 | 76.3 |
| Received treatments for COPD |  |  |  |  |  |  |
| Valve implant | 0 | 0.0 | 1 | 6.3 | 1 | 2.6 |
| Vapor ablation | 0 | 0.0 | 2 | 12.5 | 2 | 5.3 |
| Respiratory physiotherapy* | 13 | 76.5 | 10 | 62.5 | 27 | 71.1 |
| Oxygen therapy | 10 | 58.8 | 7 | 43.8 | 19 | 50 |
| Pulmonary rehabilitation (*n* = 37) | 13 | 76.5 | 14 | 93.3 | 32 | 86.5 |
| AECOPD in the past | 10 | 58.8 | 8 | 50 | 21 | 55.3 |
| Last hospitalization due to AECOPD (months)  (*M* ± *SD*; Range) (*n* = 25) | 16.62 ± 18.43;  0 - 60 | | 32.38 ± 36.86;  0 - 96 | | 32.71 ± 51.26;  0 - 240 | |
| Smoking (*n* = 37) |  |  |  |  |  |  |
| No | 1 | 5.9 | 0 | 0 | 1 | 2.7 |
| Pervious | 13 | 76.5 | 10 | 66.7 | 24 | 64.9 |
| Current (acute or chronic) | 2 | 11.8 | 3 | 20.0 | 9 | 24.3 |
| Current (very reduced or changed substance) | 1 | 5.9 | 2 | 13.3 | 3 | 8.1 |
| Years since quit smoking (*M* ± *SD*; Range) (*n* = 26) | 7.77 ± 6.24;  2 - 21 | | 7.88 ± 6.19;  0 - 17 | | 7.85 ± 5.97;  0 - 21 | |
| Years of smoking (*M* ± *SD*; Range) (*n* = 36) | 36.56 ± 9.2;  15 - 50 | | 36.87 ± 7.32;  30 - 55 | | 37.17 ± 8.81;  15 - 55 | |
| Number of cigarettes/day (*M* ± *SD*; Range) (*n* =36) | 24.81 ± 15.29;  8 - 60 | | 24.17 ± 10.07; 10 - 40 | | 23.04 ± 12.71;  3 - 60 | |
| Pack years (*M* ± *SD*; Range) (*n = 36*) | 45.88 ± 31.04; 11 - 100 | | 44.57 ± 20.61; 15 - 84 | | 42.83 ± 25.92;  6 - 100 | |
| Native language |  |  |  |  |  |  |
| German | 14 | 82.4 | 15 | 93.8 | 31 | 81.6 |
| Other | 3 | 17.6 | 1 | 6.3 | 7 | 18.4 |
| Family status |  |  |  |  |  |  |
| Single | 2 | 11.8 | 4 | 25 | 7 | 18.4 |
| Married/partnered | 8 | 47.1 | 9 | 56.3 | 19 | 50 |
| Divorced/widowed | 7 | 41.2 | 3 | 18.8 | 12 | 31.6 |
| Children | 13 | 76.5 | 12 | 75 | 29 | 76.3 |
| Living situation |  |  |  |  |  |  |
| Alone | 7 | 41.2 | 5 | 31.3 | 15 | 39.5 |
| With partner | 8 | 47.1 | 8 | 50 | 18 | 47.4 |
| Shared flat | 2 | 11.8 | 3 | 18.8 | 5 | 13.2 |
| Receiving caretaking | 7 | 41.2 | 3 | 18.8 | 12 | 31.6 |
| By family member(s) (*n* = 12) | 3 | 42.9 | 2 | 66.7 | 6 | 50 |
| By home care/nurse (*n* = 12) | 3 | 42.9 | 1 | 33.3 | 5 | 41.7 |
| By clinic outpatient service* (*n* = 12) | 1 | 14.3 | 0 | 0 | 1 | 8.3 |
| Highest education |  |  |  |  |  |  |
| Middle school | 5 | 29.4 | 3 | 18.8 | 8 | 21.1 |
| Apprenticeship | 4 | 23.5 | 7 | 43.8 | 12 | 34.2 |
| High school | 4 | 23.5 | 4 | 25 | 9 | 23.7 |
| College | 2 | 11.8 | 1 | 6.3 | 4 | 10.5 |
| Bachelor/Master | 2 | 11.8 | 1 | 6.3 | 4 | 10.5 |
| Employment |  |  |  |  |  |  |
| Employed | 1 | 5.9 | 2 | 12.5 | 3 | 7.9 |
| Self-employed | 0 | 0 | 3 | 18.8 | 3 | 7.9 |
| Retired | 13 | 76.5 | 10 | 62.5 | 26 | 68.4 |
| Unemployed | 3 | 17.6 | 1 | 6.3 | 6 | 15.8 |
| Net household income *(n = 37)* |  |  |  |  |  |  |
| <1250 € | 3 | 17.6 | 2 | 13.3 | 7 | 18.9 |
| 1250 – 1750 € | 3 | 17.6 | 4 | 26.7 | 8 | 21.6 |
| 1750 – 2250 € | 3 | 17.6 | 2 | 13.3 | 5 | 13.5 |
| 2250 – 3000 € | 6 | 35.3 | 4 | 26.7 | 11 | 29.7 |
| 3000 – 4000 € | 1 | 5.9 | 2 | 13.3 | 4 | 10.8 |
| 4000 – 5000 € | 1 | 5.9 | 1 | 6.7 | 2 | 5.4 |
| Previous contact psy-professionals |  |  |  |  |  |  |
| Yes | 10 | 58.8 | 12 | 75 | 25 | 65.8 |
| No | 7 | 41.2 | 4 | 25 | 13 | 34.2 |
| Previous experience mind-body interventions |  |  |  |  |  |  |
| Yes | 12 | 70.6 | 10 | 62.5 | 25 | 65.8 |
| No | 5 | 29.4 | 6 | 37.5 | 13 | 34.2 |
| Patient Health Questionnaire (PHQ) |  |  |  |  |  |  |
| Somatic symptoms (PHQ-15) (*M* ± *SD*; Range)  (*n* = 35) | 10.82 ± 5.87;  2 - 24 | | 9.00 ± 4.00;  3 - 16 | | 10.17 ± 5.00;  2 - 24 | |
| Depression (PHQ-9) (*M* ± *SD*; Range) (*n* = 36) | 7.35 ± 4.23;  0 - 13 | | 8.63 ± 3.88;  3 - 16 | | 7.83 ± 3.95;  0 - 16 | |
| Stress (*M* ± *SD*; Range) (*n* = 30) | 5.85 ± 3.18;  1 - 12 | | 5.69 ± 3.43;  1 - 13 | | 5.73 ± 3.06;  1 - 13 | |
| Somatoform syndrome (*n* = 37) | 7 | 41.2 | 5 | 31.3 | 14 | 37.8 |
| Major depressive disorder | 0 | 0 | 2 | 12.5 | 2 | 5.3 |
| Other depressive disorder | 4 | 23.5 | 5 | 31.3 | 10 | 26.3 |
| Panic syndrome (*n* = 37) | 1 | 5.9 | 3 | 20 | 4 | 10.8 |
| Other anxiety syndrome | 1 | 5.9 | 0 | 0 | 1 | 2.6 |
| Alcohol syndrome (*n* = 37) | 3 | 18.8 | 5 | 31.3 | 8 | 21.6 |
| *Note.* *n* = 5 dropped out before allocation. Percent are valid percent for the sample without missings. In the case of missings, the n for the valid percent is reported in brackets. *answered as "other" and has been specified by research team. BMI = body mass index, FEV = forced expiratory volume, FVC = forced vital capacity, LAMA = Long-Acting Muscarinic Antagonist, LABA = Long-Acting Beta Antagonist, ICS = Inhaled Corticosteroids. | | | | | | |

## Table S2. *Telephone interview*

|  | **Item** | **Agree** | |
| --- | --- | --- | --- |
|  |  | ***n* = 14** | **%** |
| 1 | The study was interesting. | 13 | 93 |
| 2 | People reacted to my study participation (i.e., practicing mindfulness, answering questions in app). | 4 | 29 |
| 3 | Study participation (i.e., practicing mindfulness, answering questions in app) was disturbing. | 3 | 21 |
| 4 | I often found the weekly calls to be disturbing. | 0 | 0 |
| 5 | I often found the weekly calls to be motivating. | 6 | 43 |
| 6 | I have experienced differences in daily routine and state of mind between weekdays and weekends | 4 | 29 |
| 7 | I would have been willing to make more than one data entries daily. | 11 | 79 |
| 8 | I was able to express my actual feelings and changes using the given terms. | 9 | 64 |
| 9 | I have changed my behaviour due to my study participation. | 6 | 43 |
| 10 | My study participation led to increased self-observation. | 10 | 71 |
| 11 | I participated in this study with certain expectations. | 7 | 50 |
| 12 | I have experienced special events during the study participation (e.g., loss of a loved one, lottery win). | 3 | 21 |
| 13 | I regularly took my medication during the study participation. | 14 | 100 |
| 14 | I would have needed more technical support for the app use. | 0 | 0 |
| 15 | Practicing once daily was too much for me. | 2 | 14 |
| 16 | Practicing was easier on weekends than weekdays. | 1 | 7 |
| 17 | Practicing was easier on weekdays than on weekends. | 2 | 14 |
| 18 | I often used breathing techniques (e.g., pursed lip breathing) during my mindfulness practice. | 5 | 36 |
| 19 | I mostly practiced at a specific time. | 7 | 50 |
| 20 | I mostly practiced in a specific place. | 12 | 86 |
| 21 | I mostly practiced in specific situations. | 1 | 7 |
| 22 | Had to learn many things before I could self-administer the mindfulness activities. | 0 | 0 |
| 23 | There were things that made it easier for me to perform mindfulness exercises or motivated me to practice. | 7 | 50 |
| 24 | There were things that made it more difficult for me to perform mindfulness exercises. | 6 | 43 |
| 25 | I also practiced mindfulness without using the app. | 3 | 21 |
| 26 | I integrated the mindfulness practice into my daily life. | 10 | 71 |
| 27 | I generally found the mindfulness activities helpful. | 10 | 71 |
| 28 | I generally found the mindfulness activities pleasant. | 13 | 93 |
| 29 | I noticed changes through my mindfulness practice. | 12 | 86 |
| 30 | I noticed changes in the way I deal with my lung condition through my mindfulness practice. | 3 | 21 |
| 31 | I feel like I have learned something through my mindfulness practice. | 14 | 100 |
| 32 | I plan to continue practicing mindfulness. | 13 | 93 |
| 33 | I would now like to delve more deeply into mindfulness practices and the topic mindfulness in general. | 10 | 71 |

*Note.* Data were collected during the telephone interview assessing feasibility. The interviewer noted answers to these closed questions, and they have been verified in a second step, using the audio recordings by the interviewer. Participants could elaborate on each of the discussed topics by answering to open-ended follow-up questions, which was analyzed via thematic analysis.

## Table S3. *Post monitoring questionnaire*

|  | **Item** | **Agree** | | |
| --- | --- | --- | --- | --- |
|  |  | **IG**  **(*n* = 12)** | **WL**  **(*n* = 15)** | **Total**  **(*n* = 27)** |
| 1 | The study was interesting. | 11 (92%) | 14 (93%) | 25 (93%) |
| 2 | The 6 months of study participation reflect my usual life situation. ^a^ | 9 (75%) | 12 (80%) | 21 (78%) |
| 3 | Study participation (surveys, wearing the chest belt or sensors, hair sampling) was disturbing. ^a^ | 0 (0%) | 1 (7%) | 1 (4%) |
| 4 | I often found wearing the chest belt to be disturbing. | 0 (0%) | 0 (0%) | 0 (0%) |
| 5 | I often found wearing the hand sensors to be disturbing. | 0 (0%) | 0 (0%) | 0 (0%) |
| 6 | I often found the hair sampling to be disturbing. (*n* = 26) | 0 (0%) | 2 (13%) | 2 (8%) |
| 7 | I often found the colour word test to be disturbing. (*n* = 26) | 1 (8%) | 1 (7%) | 2 (8%) |
| 8 | I often found filling out the questionnaires to be disturbing. | 1 (8%) | 0 (0%) | 1 (4%) |
| 9 | I would have been willing to wear the chest belt and hand sensors more than six times. | 11 (92%) | 13 (87%) | 24 (89%) |
| 10 | I would have been willing to fill out questionnaires more than six times. | 12 (100%) | 12 (80%) | 24 (89%) |
| 11 | I was able to express my actual feelings and changes using the given terms. ^a^ | 9 (75%) | 13 (87%) | 22 (82%) |
| 12 | I have changed my behaviour due to my study participation. ^a^ | 7 (58%) | 6 (40%) | 13 (48%) |
| 13 | My study participation led to increased self-observation. (*n* = 25) | 7 (58%) | 9 (60%) | 16 (64%) |
| 14 | I regularly took my medication during the study participation. ^a^ | 12 (100%) | 15 (100%) | 27 (100%) |
| 15 | I participated in this study with certain expectations. ^a^ | 5 (42%) | 10 (67%) | 15 (56%) |
| 16 | I have experienced special events during the study participation (e.g., loss of a loved one, lottery win). ^a^ | 2 (17%) | 11 (73%) | 13 (48%) |
| 17 | I have been vaccinated against Covid-19. ^a^ | 11 (92%) | 14 (93%) | 25 (93%) |
| 18 | I have been infected with the Covid-19 virus (at least once). ^a^ | 1 (8%) | 5 (33%) | 6 (22%) |
| 19 | I was worried about the Covid-19 situation within the last 6 months. ^a^ | 5 (42%) | 10 (67%) | 15 (56%) |
| 20 | I have been hospitalized with the last 6 months. ^a^ | 3 (25%) | 3 (20%) | 6 (22%) |
| 21 | I have had a respiratory crisis within the last 6 months. ^a^ | 5 (42%) | 6 (40%) | 11 (41%) |
| 22 | I have attended a rehabilitation program within the last 6 months. ^a^ | 2 (17%) | 1 (7%) | 3 (11%) |
| 23 | I have used psychological support services within the last 6 months (other than provided within the study). ^a^ | 0 (0%) | 1 (7%) | 1 (4%) |
| 24 | I have practiced mindfulness in the last 4 months. ^b^ (*n* = 24) | 4 (40%) | 14 (100%) | 18 (75%) |
| 25 | I have practiced mindfulness in the last 4 months, using the study activities. (*n* = 24) | 4 (40%) | 12 (86%) | 16 (67%) |
| 26 | I have practiced other mind-body techniques such as relaxation exercises, meditation, autogenic training, imaginative methods, autosuggestion, yoga, qi gong, or tai chi in the last 4 months. ^a, c^ (*n* = 24) | 1 (10%) | 2 (14%) | 3 (13%) |

*Note.* IG = intervention group, WL = waitlist control group. ^a^Participants could provide additional open answers. ^b^Practice frequency ranges from very unregularly/only when needed to daily. Most patients used the study exercises and some complemented them with additional exercises. ^c^Participants practiced yoga, autogenic training, relaxation exercises and breathing exercises.

## Table S4. *Group comparison over intervention period*

|  | **Intervention** | | **Control** | | **Two-sampe t-test** | | |
| --- | --- | --- | --- | --- | --- | --- | --- |
|  | ***M*** | ***SD*** | ***M*** | ***SD*** | ***t*** | ***df*** | ***p*** |
| **Primary Outcomes** |  |  |  |  |  |  |  |
| Anxiety (HADS-A) |  |  |  |  |  |  |  |
| Baseline | 6.86 | 2.28 | 8.06 | 3.53 | -1.12 | 25.927 | .272 |
| 4 Weeks | 6.14 | 2.74 | 9.63 | 3.83 | -2.89 | 27.026 | **.008** |
| 8 Weeks | 6.29 | 3.22 | 9.94 | 3.66 | -2.91 | 27.997 | **.007** |
| Depression (HADS-D) |  |  |  |  |  |  |  |
| Baseline | 9.71 | 3.65 | 9.00 | 4.02 | 0.51 | 27.952 | .614 |
| 4 Weeks | 8.93 | 4.25 | 9.19 | 4.72 | -0.16 | 27.969 | .876 |
| 8 Weeks | 8.79 | 4.53 | 9.69 | 4.29 | -0.56 | 26.996 | .581 |
| **Secondary Outcomes** |  |  |  |  |  |  |  |
| Self-reported stress (PSS-10) | |  |  |  |  |  |  |
| Baseline (*n* = 27) | 17.17 | 6.64 | 17.87 | 6.81 | -0.27 | 23.964 | .790 |
| 4 Weeks | 16.57 | 5.98 | 19.81 | 7.44 | -1.32 | 27.827 | .197 |
| 8 Weeks | 15.86 | 6.06 | 20.31 | 5.61 | -2.08 | 26.753 | **.047** |
| Physical health status impairment (CAT) | | |  |  |  |  |  |
| Baseline | 21.71 | 7.42 | 22.94 | 8.64 | -0.42 | 27.994 | .680 |
| 4 Weeks | 21.64 | 7.76 | 23.81 | 7.55 | -0.77 | 27.246 | .446 |
| 8 Weeks | 20.43 | 7.26 | 22.38 | 7.25 | -0.73 | 27.459 | .470 |
| Dyspnoea (CRQ-SAS) |  |  |  |  |  |  |  |
| Baseline | 3.21 | 1.15 | 4.18 | 1.42 | -2.05 | 27.857 | **.049** |
| 4 Weeks | 3.27 | 0.99 | 3.74 | 1.37 | -1.07 | 27.093 | .294 |
| 8 Weeks | 3.03 | 1.14 | 3.97 | 1.24 | -2.17 | 27.913 | **.039** |
| Fatigue (CRQ-SAS) |  |  |  |  |  |  |  |
| Baseline | 3.50 | 1.15 | 3.58 | 0.93 | -0.20 | 25.123 | .841 |
| 4 Weeks | 3.75 | 1.22 | 3.31 | 1.46 | 0.89 | 27.958 | .380 |
| 8 Weeks | 3.63 | 1.16 | 3.28 | 1.37 | 0.75 | 27.976 | .461 |
| Emotional functioning (CRQ-SAS) | |  |  |  |  |  |  |
| Baseline | 3.95 | 0.96 | 4.04 | 1.11 | -0.23 | 27.997 | .820 |
| 4 Weeks | 4.24 | 1.22 | 3.83 | 1.25 | 0.92 | 27.642 | .365 |
| 8 Weeks | 4.31 | 1.34 | 3.70 | 1.11 | 1.34 | 25.397 | .192 |
| Mastery (CRQ-SAS) |  |  |  |  |  |  |  |
| Baseline | 3.79 | 1.24 | 3.97 | 1.15 | -0.42 | 26.723 | .680 |
| 4 Weeks | 4.28 | 1.41 | 3.94 | 1.49 | 0.65 | 27.815 | .524 |
| 8 Weeks | 4.09 | 1.34 | 3.84 | 1.19 | 0.53 | 26.254 | .603 |
| Hair cortisol concentration (pg/mg) | |  |  |  |  |  |  |
| Baseline (*n* = 23) | 5.15 | 3.26 | 5.31 | 3.57 | -0.11 | 21.000 | .910 |
| 8 Weeks (*n* = 25) | 5.57 | 2.62 | 5.67 | 3.82 | -0.08 | 22.956 | .938 |
| Stress reactivity - subjective stress (VAS) | | |  |  |  |  |  |
| Baseline | 7.21 | 10.97 | 7.28 | 12.54 | -0.016 | 28.00 | .988 |
| 4 Weeks (*n* = 25) | 3.58 | 8.39 | 4.62 | 11.48 | -0.258 | 21.91 | .799 |
| 8 Weeks (*n* = 27) | -0.15 | 6.31 | 2.79 | 12.06 | -0.802 | 19.92 | .432 |
| Stress reactivity - HR (bpm) |  |  |  |  |  |  |  |
| Baseline (*n* = 28) | 4.18 | 3.35 | 5.58 | 3.66 | -1.054 | 25.80 | .301 |
| 4 Weeks (*n* = 26) | 0.76 | 0.91 | 4.35 | 3.19 | -3.896 | 13.96 | **.002** |
| 8 Weeks (*n* = 26) | 2.85 | 3.23 | 6.59 | 9.29 | -1.373 | 14.86 | .190 |
| Stress reactivity - RMSSD (ms) | |  |  |  |  |  |  |
| Baseline (*n* = 24) | 0.39 | 6.68 | 0.94 | 10.81 | -0.155 | 21.67 | .878 |
| 4 Weeks (*n* = 24) | 0.93 | 8.65 | 1.77 | 13.98 | -0.180 | 20.33 | .859 |
| 8 Weeks (*n* = 23) | 3.45 | 8.94 | 1.13 | 4.00 | 0.815 | 15.53 | .428 |
| Stress reactivity - SCL (µS) |  |  |  |  |  |  |  |
| Baseline | 2.68 | 2.29 | 4.22 | 2.27 | -1.844 | 27.41 | .076 |
| 4 Weeks (*n* = 28) | 2.35 | 2.25 | 3.34 | 2.09 | -1.203 | 24.77 | .241 |
| 8 Weeks (*n* = 27) | 2.58 | 2.31 | 3.32 | 2.17 | -0.855 | 24.52 | .401 |

Note. *N* = 30, except otherwise indicated. HADS = Hospital Anxiety and Depression Scale, PSS-10 = Perceived Stress Scale, CAT = COPD Assessment Test, CRQ-SAS = Chronic Respiratory Questionnaire Self-Administered Standardized, HR = heart rate, RMSSD = root mean square of successive difference, SCL = skin conductance level.

# References

1. Moss-Morris R, Petrie KJ. Experimental evidence for interpretive but not attention biases towards somatic information in patients with chronic fatigue syndrome. *British Journal of Health Psychology* 2003; 8: 195–208.

2. Skoluda N, Strahler J, Schlotz W, Niederberger L, Marques S, Fischer S, Thoma MV, Spoerri C, Ehlert U, Nater UM. Intra-individual psychological and physiological responses to acute laboratory stressors of different intensity. *Psychoneuroendocrinology* 2015; 51: 227–236.

3. Wilhelm S, McNally RJ, Baer L, Florin I. Directed forgetting in obsessive-compulsive disorder. *Behaviour Research and Therapy* Elsevier Ltd; 1996; 34: 633–641.

4. Becker ES, Rinck M, Margraf J, Roth WT. The emotional Stroop effect in anxiety disorders: General emotionality or disorder specificity? *Journal of Anxiety Disorders* Pergamon; 2001; 15: 147–159.

5. Creswell C, Chalder T. Underlying self-esteem in chronic fatigue syndrome. *Journal of Psychosomatic Research* 2002; 53: 755–761.

6. Tschenett H, Funk G-C, Vafai-Tabrizi F, Nater UM. Are mindfulness-based interventions feasible for patients hospitalized for acute exacerbations of chronic obstructive pulmonary disease? An exploratory interview and mixed methods study. .

7. Burdon JGW, Juniper EF, Killian KJ, Hargreave FE, Campbell EJ. The perception of breathlessness in asthma. *American Review of Respiratory Disease* 1982; 126: 825–828.

8. Borg G. Psychophysical bases of perceived exertion. *Medicine & Science in Sports & Exercise* 1982; 14: 377-381-377–381.

9. Zigmond AS, Snaith RP. The Hospital Anxiety and Depression Scale. *Acta Psychiatrica Scandinavica* John Wiley & Sons, Ltd; 1983; 67: 361–370.

10. Steyer R, Schwenkmezger P, Notz P, Eid M. Mehrdimensionaler Befindlichkeitsfragehogen (MDBF). Göttingen: Hogrefe; 1997.

11. Feneberg AC, Nater UM. An ecological momentary music intervention for the reduction of acute stress in daily life: A mixed methods feasibility study. *Front. Psychol.* 2022; 13: 927705.

12. Braun V, Clarke V. Using thematic analysis in psychology. *Qualitative Research in Psychology* 2006; 3: 77–101.

13. García-Pérez MA. Use and misuse of corrections for multiple testing. *Methods in Psychology* 2023; 8: 100120.

14. Woltman H, Feldstain A, MacKay JC, Rocchi M. An introduction to hierarchical linear modeling. *TQMP* 2012; 8: 52–69.

15. Bates D, Mächler M, Bolker B, Walker S. Fitting Linear Mixed-Effects Models Using **lme4**. *J. Stat. Soft.* [Internet] 2015 [cited 2024 Mar 5]; 67Available from: http://www.jstatsoft.org/v67/i01/.

16. Lüdecke D, Ben-Shachar M, Patil I, Waggoner P, Makowski D. performance: An R Package for Assessment, Comparison and Testing of Statistical Models. *JOSS* 2021; 6: 3139.

17. Ben-Shachar M, Lüdecke D, Makowski D. effectsize: Estimation of Effect Size Indices and Standardized Parameters. *JOSS* 2020; 5: 2815.

18. Kenward MG, Roger JH. Small Sample Inference for Fixed Effects from Restricted Maximum Likelihood. *Biometrics* 1997; 53: 983.

19. Schielzeth H, Dingemanse NJ, Nakagawa S, Westneat DF, Allegue H, Teplitsky C, Réale D, Dochtermann NA, Garamszegi LZ, Araya‐Ajoy YG. Robustness of linear mixed‐effects models to violations of distributional assumptions. Sutherland C, editor. *Methods Ecol Evol* 2020; 11: 1141–1152.

20. Koller M. **robustlmm** : An *R* Package for Robust Estimation of Linear Mixed-Effects Models. *J. Stat. Soft.* [Internet] 2016 [cited 2024 Mar 5]; 75Available from: http://www.jstatsoft.org/v75/i06/.

21. Stalder T, Steudte S, Alexander N, Miller R, Gao W, Dettenborn L, Kirschbaum C. Cortisol in hair, body mass index and stress-related measures. *Biological Psychology* 2012; 90: 218–223.
